# Family-Aware Genome-Wide Association Mapping of Growth and Body-Shape Traits in Bighead Carp (*Hypophthalmichthys nobilis*)

**DOI:** 10.3390/ani16203271

**Published:** 2026-10-17

**Authors:** Geng Chen, Fangyuan Peng, Dan Wang, Jin Xu

**Affiliations:** Yangtze River Fisheries Research Institute, Chinese Academy of Fishery Sciences, Wuhan 430223, China

**Keywords:** bighead carp, *Hypophthalmichthys nobilis*, genotyping-by-sequencing, genotype recall, imputation, full-sib families, genome-wide association study, body shape, growth

## Abstract

Reliable marker–trait associations can support genetic improvement of growth and body conformation in bighead carp (*Hypophthalmichthys nobilis*). We investigated eight traits in 569 fish assigned to 34 inferred consensus full-sib groups. Genotypes at 89,684 reference-consistent SNP positions were recalled from sequencing alignments and filtered by read depth, genotype quality, and heterozygote-allele balance. Common-mask validation of 274,566 genotype cells supported a 60% pre-imputation call-rate threshold, achieving 94.17% overall, 90.26% non-reference, and 89.04% heterozygote concordance, with hard-dosage *r*^2^ = 0.859. The final imputed matrix contained 32,304 SNPs; 9086 LD-pruned SNPs were used to estimate population structure and genomic covariance. Primary mixed-model scans used recalled genotype inputs before Beagle imputation and identified 18 suggestive trait–marker combinations. Candidate analyses using actual observed genotypes supported 17 combinations at within-group permutation FDR < 0.05, representing 16 markers. Grouping, model, and group-omission sensitivity analyses characterized their robustness. Prioritized regions contained developmentally relevant genes, including *sulf1*, *dmrt2*, *cyp24a1*, *chst12*, *efcab7*, and *anapc7*. These family-supported candidates provide targets for replication and functional investigation and may inform genetic improvement and new-variety development in bighead carp.

## Simple Summary

Improving growth and body shape is an important goal in bighead-carp breeding. We studied eight growth and morphological traits in 569 fish from 34 family groups inferred from genetic data. Sequencing records were re-examined to improve the reliability of genetic markers, and missing genotypes were estimated using a procedure evaluated against observed genotypes. Association analyses identified 18 combinations of traits and markers for further assessment. Seventeen combinations, involving 16 markers, showed support when comparisons were made within the inferred family groups. Nearby genes included *sulf1*, *dmrt2*, *cyp24a1*, *chst12*, *efcab7*, and *anapc7*, which provide biological hypotheses concerning development, tissue growth, and body conformation. These findings identify promising genomic regions for further investigation and provide a basis for evaluating genetic markers in additional breeding populations. Such work may contribute to the development of improved bighead carp varieties with desirable growth and morphological characteristics.

## 1. Introduction

Aquaculture breeding must improve production efficiency while maintaining robustness and genetic diversity under changing production environments. Selective breeding has delivered substantial gains in aquatic species, and genomic information can support breeding decisions through improved relationship estimates, estimation of breeding values, and investigation of economically important traits [1,2,3,4,5,6]. Bighead carp (*Hypophthalmichthys nobilis*) is an important freshwater aquaculture species: FAO reports Chinese production of approximately 3.86 million tons in 2023 [7]. Its head is a valued food product in Chinese markets, providing a species-specific motivation for investigating head morphology alongside growth [8]. Growth and morphological variation nevertheless reflect complex interactions among genetic architecture, developmental allometry, husbandry conditions, and family backgrounds.

Previous genetic studies have established a foundation for investigating growth and morphology in bighead carp. High-density linkage mapping identified QTL for body weight, body length, head length, and body height, including regions shared among several growth measurements [9]. Subsequent GWAS specifically examined head dimensions and proportional head-shape traits [8], while another study identified regions associated with body weight, body length, body height, and deheaded body length and investigated *fndc5b* as a candidate growth-related gene [10]. These findings demonstrate that both overall growth and head morphology have identifiable genetic components, while also motivating further assessment of candidate associations in populations with different genetic backgrounds and family structures.

Related cyprinids provide additional evidence for the genetic complexity of these traits. In silver carp, linkage mapping identified growth-related QTL for body weight, body length, body height, and head length across different ages, with some intervals shared across developmental stages [11]. In common carp, combined GWAS and linkage analysis identified head-size loci and implicated candidates associated with growth and skeletal development, including *igf1* and *igf3* [12]. In grass carp, GWAS identified associations for body weight, body length, body height, and body depth, including markers associated with multiple traits [13]. Together, these studies support examining overall growth alongside anatomically distinct morphological measurements. However, differences in phenotype definitions, developmental stages, genetic backgrounds, and mapping resources mean that findings across studies require careful comparison before they can be considered evidence for the same underlying locus.

The eight traits examined here were selected to characterize overall growth, external conformation, and a specialized component of head anatomy. Body weight and body length characterize overall growth, whereas body height describes body conformation. Head length, head width, head height, and head thickness capture complementary dimensions of a commercially valued anatomical region [8]. These measurements provide a basis for investigating morphological variation, but they are not direct measurements of head yield, fillet yield, or market price. Palatal-organ weight (PFW) characterizes a substantial, filter-feeding-related structure within the head; previous transcriptomic work documented developmental changes in this organ [14]. PFW was therefore included as an anatomical developmental trait. Allometric adjustment of the morphological measurements allowed us to examine proportional variation beyond overall size, while retaining BW and BL as indicators of general growth.

Genome-wide association studies can identify marker–trait relationships and nominate genomic regions for biological investigation, but their interpretation requires attention to population structure, relatedness, and genotype uncertainty [15,16]. In family-structured aquaculture cohorts, candidate evaluation should also examine whether genotype–phenotype correspondence is supported within groups and how strongly the estimated effect depends on particular groups.

Genotyping-by-sequencing (GBS) provides an affordable means of collecting genome-wide markers in aquaculture populations [17,18,19,20], but genotype reliability depends on read depth, library representation, and alignment quality. At low depth, sampling only one allele of a heterozygote can produce an incorrect homozygous call. Restriction-site polymorphisms can additionally cause allele-specific dropout, a distinct mechanism described in RADseq studies [21,22,23]. These limitations motivate evaluating both missingness and the reliability of retained genotype calls.

Evidence from breeding populations demonstrates the potential of imputation while identifying important conditions for its success. In Atlantic salmon, low-density marker imputation with genotyped parents supported cost-efficient genomic prediction, with imputation accuracy depending on marker density [24]. This setting differs from low-coverage sequencing, where the input genotypes themselves may be uncertain. In rainbow trout breeding populations, GLIMPSE2 imputation using a haplotype reference panel achieved high concordance with SNP-array genotypes across several sequencing depths, with concordance decreasing as coverage was reduced [25]. In large pig pedigrees, hybrid peeling enabled accurate sequence imputation by combining sequence, marker, and pedigree information; performance depended on pedigree connectedness and the availability of information from relatives [26]. These findings support imputation as a useful breeding resource, while emphasizing that accuracy must be evaluated in the population and data setting under study.

Methods for low-coverage sequencing also differ in their treatment of uncertainty and reference information. Genotype-likelihood-based approaches retain uncertainty in sequencing-derived genotypes rather than relying solely on discrete calls, whereas STITCH infers haplotypes from sequencing reads without an external haplotype reference panel [25,27]. In the present study, Beagle imputation used filtered, recalled genotype calls and within-cohort haplotype information, without an external reference panel or explicit pedigree constraints. Masking high-confidence observed calls provided an empirical assessment of genotype recovery under the selected validation conditions. Because this assessment does not establish accuracy for every originally missing or erroneous genotype, candidate evaluation additionally used read-level quality information and actual observed candidate genotypes.

Family structure creates a second, partly independent challenge. In populations composed of full-sib groups, allele-frequency differences and phenotype differences can align between families, while only a subset of families may segregate at any given locus. Principal components and genomic-relationship matrices are powerful tools for controlling broad structure [28,29,30,31], but mixed-model performance depends on the genetic architecture, sampling design, and relationship between the tested marker and the covariance model [32,33,34,35]. Permutations that preserve family membership and effect estimates based on within-family contrasts address a sharper question: whether genotype–phenotype correspondence remains after between-family differences have been removed [36]. Leave-one-family-out analysis then tests whether the conclusion is broadly distributed or driven by one influential cross.

Separating overall size from proportional morphology changes the interpretation of growth and body-shape associations [37,38]. The primary objective of this study was to identify suggestive marker associations with overall growth and size-adjusted morphological traits in a communally reared, family-structured bighead carp cohort. We hypothesized that some associations would retain support within inferred sibship groups after accounting for differences among groups. To evaluate this hypothesis, we combined genotype recall and imputation assessment with candidate analyses using actual observed genotypes, alternative groupings, and association-model sensitivity tests. Candidate regions were annotated and examined using public expression data to generate biological hypotheses. Incremental phenotypic variance and exploratory group-held-out prediction analyses were also used to assess potential breeding relevance. Together, these analyses aimed to prioritize candidates for replication and functional investigation and provide evidence relevant to future improvement of growth and body conformation in bighead carp.

## 2. Materials and Methods

### 2.1. Animals, Family Population, and Phenotyping

The starting population comprised 598 bighead carp obtained from Liangzi Lake Experimental Farm, Yangtze River Fisheries Research Institute, Chinese Academy of Fishery Sciences, Wuhan, China. The fish were produced in May 2025 through a complete factorial mating design involving six female and six male broodstock. Eggs from each of the six females were artificially fertilized separately with sperm from each of the six males, producing 36 planned cross combinations. Fertilized eggs from all crosses were pooled before hatching, and the offspring were reared communally. At 28 days post-hatching, 2000 fry from this pooled cohort were transferred to a single pond for continued communal rearing. During the growing season, water temperature ranged from approximately 22 to 30 °C, and dissolved oxygen ranged from 4.3 to 6.7 mg/L. Bighead carp are filter-feeding fish, and no artificial feed was provided.

Breeding and pond rearing formed part of routine production at the institute’s Liangzi Lake Experimental Farm. In May 2026, 598 yearling fish were randomly sampled following seine netting of the communally reared cohort, and research phenotyping and sampling were performed after ethical approval. Sex was not determined. All 598 fish were sequenced. During sample-level filtering of the initial R50_DP2 dataset containing 90,821 SNPs, eight individuals with genotype missingness greater than 60% were excluded, leaving 590 individuals. Subsequent sibship-consensus filtering excluded 17 individuals unassigned in the primary reconstruction and four with insufficient cross-model support, yielding a final analysis cohort of 569 fish. Anaesthesia and euthanasia were performed using MS-222. Linear dimensions were measured with a digital vernier caliper (resolution, 0.01 mm). Body weight and palatal-organ weight were measured using electronic balances with resolutions of 0.1 g and 0.01 g, respectively, according to the laboratory measurement protocol. The recorded traits were body weight (BW), body length (BL), head length (HL), body height (BH), head height (HH), head thickness (HT), palatal-organ weight (PFW), and head width (HW). Measurements were checked for biologically implausible cross-trait combinations.

Phenotype screening used outer Tukey fences (Q1 − 3 × IQR and Q3 + 3 × IQR), absolute median/MAD-based robust *z*-scores greater than 3.5, and studentized residuals from log–log allometric models. These diagnostics were used to identify measurements for assessment rather than as automatic exclusion rules. The final masking set was a documented, case-by-case selection based on discordance among measurements from the same fish. Eleven measurements in eleven fish were set to missing: two BL values inconsistent with multiple other traits; two HL, one BH, two HH, and two HT values inconsistent with BL; one PFW value inconsistent with BL, BW, and HW; and one HW value inconsistent with BW and BL. Other measurements from these fish were retained. Sample identifiers, original values, and masking reasons are reported in Table S1a. The protocol for the present study was approved by the Ethics Committee of the Yangtze River Fisheries Research Institute, Chinese Academy of Fishery Sciences, on 10 January 2026 (protocol code YFI2026110).

### 2.2. Phenotype Transformation and Allometric Adjustment

Positive measurements were natural-log transformed. Overall growth was represented by logBW and logBL. For relative traits, ordinary least-squares models of the form log(Y) = α + γ log(S) + ε were fitted across the pooled cohort, where Y denotes the response trait and S its size covariate. BL was used for HL, BH, HH, and HT; BW for HW; and HW for PFW. These specifications define variation conditional on body length, body weight, or HW, respectively. Model residuals were used as the six adjusted GWAS phenotypes. Each model included only fish with both measurements available. The original allometric models did not include inferred group effects; their sample sizes, slopes, and fit statistics are reported in Table S1d.

To assess sensitivity to this two-stage adjustment, the 18 selected candidate trait–marker combinations were additionally evaluated using one-stage linear models. For the six adjusted traits, these models jointly included the original log-transformed response, observed candidate genotype dosage, the corresponding log-transformed size covariate, and inferred group fixed effects. For logBW and logBL, the original group-fixed model specification was retained. Each comparison used identical samples and genotype coding, and reproduction of the original group-fixed coefficient was verified before comparison. Two-sided coefficient tests were adjusted across the 18 combinations using the Benjamini–Hochberg procedure. These parametric tests were reported separately from the original within-group permutation tests.

### 2.3. Family Assignment and Family-Level Phenotypic Variance

Sibship groups were inferred using sequoia v3.2.0 from non-imputed GBS genotypes [39]. The primary reconstruction used 590 offspring and 900 SNPs distributed across all 24 linkage groups. Offspring sex was coded as unknown, and all offspring were assigned to the same birth cohort. Reconstruction used Module = “ped”, Err = 0.02, ErrFlavour = “version2.9”, Tfilter = −2, Tassign = 0.5, MaxSibshipSize = 590, Complex = “simp”, Herm = “no”, UseAge = “yes”, and StrictGenoCheck = FALSE. A group was defined by a shared pair of inferred dummy-parent identifiers. These identifiers represent latent parental assignments and were not matched to the actual broodstock.

The primary reconstruction assigned two inferred parents to 573 offspring, forming 35 parent-pair groups. Assignment stability was evaluated against three sensitivity reconstructions of the same offspring cohort: “panel451_E02”, using a 451-SNP panel with Err = 0.02; “E01”, using the 900-SNP panel with Err = 0.01; and “RAD”, using the 900-SNP panel with the error model generated by Err_RADseq(E0 = 0.005, E1 = 0.05). Alternative groups were mapped to the primary groups by overlap. A mapping contributed to consensus voting only when it was unambiguous and had mapping purity ≥ 0.80. An offspring assignment was retained when at least two of the three sensitivity reconstructions supported its primary group. This procedure retained 569 offspring in 34 inferred consensus full-sib groups and excluded 17 offspring unassigned in the primary reconstruction and four with insufficient cross-model support. Retained consensus-group sizes ranged from two to eighty-seven offspring. Group membership was distinguished from marker-specific informativeness, which depended on the available observed genotypes, phenotypes, and within-group genotype variation. Sample-level assignments, group sizes, and exclusion reasons are reported in Table S2. The 34 retained groups represent the inferred partition of the analyzed offspring and should not be interpreted as a verified count of the 36 planned crosses. To assess grouping stability using complementary genotype evidence, we constructed an additional panel from non-imputed recalled genotypes, excluding SNP coordinates represented in the original 900-SNP panel. Genotype calls were required to have DP ≥ 4 and GQ ≥ 20, and retained sites were required to have MAF ≥ 0.15 and a post-filtering call rate ≥ 0.60. Greedy pruning applied a minimum spacing of 20 kb and a local genotype-correlation threshold of r^2^ ≤ 0.20 within 1 Mb, retaining 1312 SNPs across all 24 linkage groups. Joint sibship reconstruction was repeated from scratch under the E02 and RAD error models. Agreement with the original grouping was quantified using the adjusted Rand index among offspring assigned two inferred parents. This complementary panel provided additional marker evidence within the same cohort, rather than independent-population validation.

All 18 candidate trait–marker tests were subsequently reanalyzed under four datasets: the original 569-offspring grouping, the RAD-derived grouping of the same 569 offspring, the original grouping restricted to the 565 offspring assigned under E02, and the E02-derived grouping of those same 565 offspring. The matched 565-offspring comparison separated changes in grouping from changes caused by sample exclusion. Phenotype values and candidate genotype calls were retained from the original analyses. Family fixed-effect estimation, 10,000 within-group permutations, Benjamini–Hochberg adjustment across the 18 tests within each analysis, and leave-one-group-out diagnostics followed the original procedures (Table S12).

For each transformed phenotype, we fitted a random-intercept model with family as the grouping factor using lme4 [40]. For relative traits, the size covariate used during allometric adjustment was included in the model definition. Family intraclass correlation was calculated as ICC = σ^2^_family_/(σ^2^_family_ + σ^2^_residual_). Confidence intervals were obtained from 1000 bootstrap replicates. The family effect was tested using a variance-component likelihood-ratio statistic with 2000 bootstrap replicates; the resulting *p* values were adjusted across the eight traits by the Benjamini–Hochberg procedure [41].

Tfilter is the log10-likelihood-ratio threshold used to screen candidate relatives against the unrelated relationship, whereas Tassign is the minimum log10-likelihood-ratio threshold for accepting a proposed relationship relative to the most likely alternative.

Pairwise relationship support was assessed using sequoia’s CalcPairLL() function with the complementary 1312-SNP observed-genotype panel under the E02 and RAD error models. Within-group and between-group pairs were summarized separately. A pair was classified as supporting full siblings when the full-sibling relationship had the highest log10-likelihood and exceeded the best alternative relationship by at least 0.5 log10 units. These classifications were used as model-based diagnostics of sibship support, rather than direct verification of broodstock identities.

### 2.4. GBS Variant Set and Reference-Allele Harmonization

Genomic DNA was extracted from caudal fin clips using a phenol-chloroform protocol. GBS libraries were prepared following the protocol of Elshire et al. [18] with minor modifications. Briefly, genomic DNA was digested with ApeKI (New England Biolabs, Ipswich, MA, USA) at 75 °C, followed by ligation of barcoded adapters, PCR amplification, and size selection. Libraries were sequenced on the Illumina NovaSeq 6000 platform (Illumina, San Diego, CA, USA) with paired-end 150 bp (PE150) reads at Annoroad Gene Technology Co., Ltd. (Beijing, China).

Quality-controlled paired-end reads were aligned to the bighead carp reference assembly Hypophthalmichthys_nobilis_1.0 [42] using BWA-MEM2 v2.2.1 with the -M option and sample-specific read groups [43]. Alignments were sorted by genomic coordinate using SAMtools v1.24 [44].

Initial SNP discovery and genotyping were performed using Stacks v2.68 in reference-based mode [45]. The gstacks program used the marukilow model, with variant-discovery and genotype-calling alpha thresholds of 0.05, a minimum mapping quality of 20, a maximum soft-clipping fraction of 0.20, and a maximum insert length of 1000 bp. The populations program required locus presence in at least 50% of samples (-R 0.50), a minimum genotype depth of two reads, a minor-allele frequency ≥0.01, and an observed heterozygosity ≤ 0.70; variants were exported using --ordered-export. This initial panel contained 90,821 SNPs in 598 sequenced fish. Eight individuals with genotype missingness >60% in this panel were excluded. Genotype calls with GQ < 10 were subsequently set to missing using bcftools v1.24 [44]. Following sample and site quality control, the candidate-site catalog contained 89,807 SNPs in 590 fish.

Across all 598 sequenced fish, gstacks reported a mean effective per-sample coverage of 2.5× (standard deviation, 0.6×; range, 1.7–6.3×). Separately, the median of the per-sample mean DP values recorded for the initial SNP panel was 3.85 (range, 2.97–10.34). Among the 590 retained fish, the corresponding median was 3.86 (range, 3.15–10.34). The gstacks effective-coverage estimate and SNP-panel DP summaries represent different coverage measures. The initial Stacks-derived catalog provided the candidate positions for subsequent reference-allele harmonization and BAM-based genotype recall.

We retained 89,773 sites on the expected 24 linkage groups and removed 34 records on four unplaced or malformed contigs. Marker identifiers were standardized as chromosome:position:reference:first-alternate. Duplicate coordinates, duplicate variants, and duplicate marker identifiers were absent.

Reference alleles were compared with the bighead carp genome sequence using bcftools norm [44]. Of 89,773 linkage-group sites, 6955 were simple REF/ALT swaps and were corrected while preserving genotype meaning; 89 non-simple mismatches (42 strand-flip swaps, 23 strand-flip keeps, and 24 unresolved mismatches) were excluded. The resulting 89,684-site VCF passed a second strict reference check. The source genotype records contained GT, DP, and GQ for every called genotype (Table S3).

### 2.5. BAM-Based Genotype Recall and Cell-Level Filtering

To reduce dependence on the original caller, all 89,684 positions were recalled from the 569 analysis BAM files. For each linkage group, bcftools mpileup used minimum mapping quality 20, minimum base quality 20, a per-file maximum depth of 1000, and FORMAT/AD, ADF, ADR, DP, and SP annotations. Alleles were forced to the reference-consistent biallelic definition before multiallelic calling. Per-genotype masking retained a called genotype only when DP ≥ 2 and GQ ≥ 10; heterozygotes additionally required minor allele balance ≥ 0.20. AC, AN, AF, and F_MISSING were recalculated after masking.

Depth distributions were summarized per sample and per site across all 89,684 targets, including zero-depth cells. Sites without a passing genotype were classified according to recorded depth, genotype quality, and raw genotype calls. Missing GQ values failed the quality filter.

An internal read-downsampling benchmark used 8640 genotype-balanced reference cells from 96 fish selected across the ranked per-sample mean depths. Reference calls required DP ≥ 12, GQ ≥ 30 and total allelic depth ≥ 12, with minor allele balance ≥ 0.30 for heterozygotes and called-allele fraction ≥ 0.95 for homozygotes. Read templates were subsampled at fractions of 0.125, 0.25, and 0.50 in three repetitions. All 569 fish were jointly recalled using the original calling parameters and target definitions. The full-read control reproduced all reference genotypes and depths. Performance was stratified by realized DP and compared across DP/GQ thresholds of 2/10, 4/20, and 6/30, retaining the heterozygote allele-balance threshold of 0.20. Concordance was calculated among retained calls, whereas retention used all reference-cell evaluations in the stratum as the denominator. This benchmark assessed internal concordance against full-read calls from the same sequencing data (Table S4).

The recalled dataset contained 53,935 polymorphic sites, 233 all-reference sites, 66 fixed-alternate sites, and 35,450 sites without any genotype passing cell filters. The overall observed cell call rate was 0.490. Candidate-site sets were defined by MAF ≥ 0.05 and observed call-rate thresholds of 0.50, 0.60, or 0.70, yielding 32,724, 32,417, and 30,095 SNPs, respectively (Table S4).

### 2.6. Masking Validation and Genotype Imputation

High-confidence observed cells (DP ≥ 4 and GQ ≥ 30) were stratified by linkage group and true genotype. Five percent of eligible cells were masked reproducibly with seed 20260920. To make the call-rate comparison fair, the same 274,566 truth cells were masked in all three site sets. Each masked dataset was phased and imputed with Beagle 5.5 [46,47] using effective population size 500, allele-mismatch probability 0.001, EM parameter estimation disabled, and seed 20260920. No genetic map was available, so Beagle used its default physical-to-genetic distance conversion.

Evaluation used hard GT because Beagle emitted GT without per-genotype DS/GP at sites already present in the study-only input. Metrics included imputation rate, overall and genotype-specific concordance, non-reference concordance, balanced accuracy, Cohen’s kappa, hard-dosage correlation and *r*^2^, RMSE, and MAE. Concordance was additionally summarized by the original depth of the masked reference genotypes using DP strata of 4–5, 6–9, 10–19, and ≥20, both overall and separately by genotype class. The same 274,566 reference cells were evaluated in all three scenarios. Because reference-cell eligibility required DP ≥ 4 and GQ ≥ 30, this assessment did not include reference genotypes with DP < 4. CALL60 was selected as a practical compromise between marker retention and imputation performance, with marginally higher overall, non-reference, and heterozygote concordance. Final imputation of this set produced 32,304 polymorphic SNPs in 569 fish with no missing genotypes and MAF ≥ 0.05 (Table S5; Figure S2).

### 2.7. Population Structure, Linkage Disequilibrium, and Kinship

The complete Beagle-imputed panel of 32,304 SNPs was converted to PLINK format. LD pruning using PLINK 2.0 [48,49] retained 9086 SNPs for calculation of 20 principal components and the global genomic-relationship matrix. The proportion of variance in each PC explained by inferred consensus group was quantified using η^2^ from a one-way model. The global centered matrix was calculated using GEMMA 0.98.5 [31] with -gk 1 and supplied to both the primary recalled-input GWAS and the comparative Beagle-imputed analyses. It was defined as

(1) $$K_{\mathrm{ij}}^{\left(c\right)}=\frac{1}{M}\sum_{m=1}^{M}\left(x_{\mathrm{im}}-{\bar{x}}_{m}\right)\left(x_{\mathrm{jm}}-{\bar{x}}_{m}\right)$$

where M = 9086, *x_im_* is the allele dosage for offspring *i* at marker *m*, and ${\bar{x}}_{m}\;$ is the mean dosage across the 569 offspring. Sample identifiers were aligned to the matrix order before summarizing within-group and between-group off-diagonal entries. The matrix was 569 × 569, finite, and symmetric. For diagnostic comparison, a standardized matrix was additionally calculated using -gk 2 with the same markers and offspring, dividing each marker contribution by its sample genotype variance. The centered matrix was retained for all primary association models. Matrix entries were interpreted as sample-relative genomic relationships rather than broodstock-verified pedigree coefficients. Because genome-wide LD pruning and GRM construction used Beagle-imputed genotypes, the resulting LD estimates and genomic relationships may be affected by imputation; LD pruning reduces marker redundancy but does not eliminate potential imputation-related effects.

### 2.8. Genome-Wide Association Analysis

The primary GWAS used the CALL60 recalled genotype dataset before Beagle imputation, containing 32,417 SNPs with MAF ≥ 0.05 and an observed call rate ≥ 0.60. Each of the eight transformed phenotypes was analyzed using a univariate linear mixed model in GEMMA 0.98.5 [31], with the global centered genomic-relationship matrix described in Section 2.7. The primary PC0 specification included an intercept but no additional PC covariates. The GEMMA command specified -miss 0.40, -maf 0.05, and -lmm 4; the likelihood-ratio test *p* value was used for association ranking.

For retained SNPs, GEMMA replaced missing genotype entries with the SNP-specific mean. Individuals with missing phenotypes were excluded from the corresponding trait analysis. Accordingly, the primary scan used recalled genotype inputs without Beagle haplotype imputation, but was not a complete-case analysis restricted to individuals with an observed genotype at every tested SNP. Candidate-level analyses restricted to actual observed genotypes are described separately in Section 2.9.

Comparative GWAS analyses used the final Beagle-imputed dataset under PC0, PC3, and PC5 specifications, corresponding to 24 trait-model combinations. PC3 and PC5 included the first three and five PCs, respectively, calculated from the LD-pruned imputed dataset. These comparative analyses were distinguished from the primary recalled-genotype PC0 scans. For each analysis with M-tested SNPs, the Bonferroni and suggestive thresholds were defined as 0.05/M and 1/M, respectively. Genomic inflation factors and quantile–quantile plots were examined, and results were reported separately by genotype dataset and model specification (Table S6; Figure S1).

To assess sensitivity to proximal contamination, we additionally performed leave-one-linkage-group-out (LOCO) analyses [35]. For each of the 24 linkage groups, a centered GRM was calculated with GEMMA (-gk 1) using the original LD-pruned SNP set after excluding all markers on that linkage group. The LD-pruned set was derived from the final imputed matrix, whereas association tests retained the same recalled genotype inputs before Beagle imputation as the primary analyses. Phenotypes, intercept-only covariates, allele coding, MAF and missingness filters, and GEMMA’s internal replacement of missing genotypes were kept unchanged. The 24 linkage-group-specific scans were combined for each trait. Effect estimates, likelihood-ratio *p* values, and genomic-inflation factors were compared with the global-GRM results. Bonferroni and suggestive thresholds were calculated from the total number of tested markers across all linkage groups for each trait (Table S13).

PC0 was retained as the primary specification used in the original analysis, with PC3 and PC5 evaluated as sensitivity models. To assess PC sensitivity directly in the primary observed-input analysis, all eight traits were additionally analyzed with the first three or first five PCs as covariates. Within each trait, genotype inputs, phenotypes, sample sets, tested markers, effect-allele coding, filtering settings, and the global genomic-relationship matrix were held constant. These comparisons were distinguished from the imputed-input PC grid. Model sensitivity was assessed using genomic inflation factors, candidate-effect estimates and directions, and retention of suggestive and Bonferroni support (Table S14). Fourteen nonfinite likelihood-ratio *p* values in the logBL PC5 scan were excluded from *p*-based diagnostic summaries but retained in the tested-marker denominator used to calculate association thresholds; none involved an original candidate (Table S14c).

### 2.9. Candidate-Locus Validation

Candidate trait–marker combinations were selected when their likelihood-ratio *p* values were below the trait-specific suggestive threshold of 1/M in the primary recalled-input PC0 scans, where M was the number of SNPs tested for that trait. All combinations meeting this rule were retained for candidate-level assessment, yielding 18 tests at 17 unique markers. Candidate loci were audited using the recalled genotype records before Beagle imputation. Technical summaries included observed call rate, MAF, genotype counts, DP, GQ, heterozygote-allele balance, forward/reverse allele fractions, the number of inferred consensus groups carrying the minor allele, and the number showing within-group genotype variation. For each candidate trait–marker combination, family-fixed effect estimation, within-group permutation, and leave-one-group-out analysis used actual observed genotypes and the corresponding available phenotypes. Genotypes were coded as alternate-allele counts of 0, 1, or 2; missing genotype or phenotype records were excluded from these candidate-level datasets. Neither Beagle-imputed calls nor GEMMA mean-replaced values were included. Family-fixed models estimated the genotype coefficient conditional on inferred consensus group.

For the within-group permutation test, genotype dosage and phenotype were centered by subtracting their respective group means. The test statistic was the absolute within-group genotype-effect coefficient, calculated as the sum of the products of centered dosage and phenotype divided by the sum of squared centered dosage. Genotypes were permuted within inferred consensus groups 10,000 times while retaining the observed complete-case sample set and group membership. Two-sided empirical *p* values were calculated as (1 + B)/(10,001), where B was the number of permutations with an absolute coefficient at least as large as the observed value. The Benjamini–Hochberg procedure was applied across the 18 selected trait–marker tests [41]. These candidate-level permutation results do not account for marker selection across the preceding genome-wide scan.

Group-specific dosage effects were estimated using actual observed genotypes and available transformed phenotypes. Sample sizes, genotype counts, standard errors, and *t*-based 95% confidence intervals were reported; standard errors and confidence intervals were left unavailable when residual degrees of freedom were insufficient. Heterogeneity was evaluated using a nested *F* test comparing a common dosage slope with separate slopes for informative groups, conditional on group intercepts and using identical complete-case observations. Exact two-sided binomial sign tests compared the numbers of positive and negative group-specific slopes, excluding exactly zero slopes. Heterogeneity and sign-test *p* values were separately BH-adjusted across the 18 candidates.

Internal effect stability was assessed using 100 repeated splits of the 34 inferred groups into two complementary halves. Groups were paired by cohort size, with one group from each pair randomly allocated to each half, yielding 17 intact groups per half. The same splits were used for all candidates. Group-adjusted effects were estimated separately in each half. Directional holdout tests were one-sided and used the effect direction estimated in the opposite half; simultaneous nominal support was summarized separately as two-sided *p* < 0.05 in both halves. Nonestimable halves were recorded explicitly, and proportions were calculated among evaluable splits. These summaries were descriptive because repeated splits overlapped, candidates were selected using the full cohort, and inferred groups could share parents.

Leave-one-family-out (LOFO) analysis sequentially omitted each inferred consensus group and refitted the group-fixed model. Among refits yielding finite genotype coefficients, direction consistency was the fraction retaining the sign of the full-data coefficient. The maximum absolute relative-coefficient change was calculated as max(|β_−g_ − β_full_|/|β_full_|), where β_−g_ was the coefficient after omitting group g. Tests were classified as robust when direction consistency was 100% and the maximum change was ≤25%; tests not meeting this criterion were classified as moderately robust when direction consistency was ≥90% and the maximum change was ≤50%. Remaining tests were classified as family-sensitive. These operational categories describe sensitivity to group omission. Pairwise LD and conditional models were used to assess dependence among candidate markers (Tables S7–S10; Figures S3 and S5).

For retained candidate markers on the same linkage group, pairwise LD was summarized using squared correlations between observed genotype dosages and absolute D′ calculated from Beagle-derived phases. Phased analyses included only individuals with actual observed genotypes at the relevant markers and exact agreement between observed and phased genotype dosages. All 15 pairs among the six LG04 markers were examined. Exploratory six-marker haplotype configurations were summarized among individuals with observed genotypes at all six positions. Haplotypes with frequencies below 5% were pooled, and group-adjusted omnibus linear-model tests compared models with and without haplotype-dosage terms. Six-marker conditional models included inferred group and all six observed marker dosages; corresponding marginal models used identical trait-specific samples. These analyses evaluated dependence among candidates rather than establishing independent causal effects (Table S10).

For each screened trait–marker association, incremental phenotypic variance explained (PVE) was estimated by comparing a group-only model with a group-plus-genotype model fitted to identical observed-genotype complete-case samples. Incremental PVE was calculated as 100 × (SSE_reduced − SSE_full)/SST, where SSE denotes residual sum of squares and SST denotes the total phenotypic sum of squares. Partial within-group R^2^ was reported separately using SSE_reduced as the denominator. These discovery-cohort estimates were calculated separately for each marker and were not summed across loci (Table S15a).

Phenotype prediction was assessed using leave-one-consensus-group-out cross-validation across the 34 original consensus groups. For each trait, GBLUP using the complete 32,304-SNP Beagle-imputed panel was compared with GBLUP excluding the corresponding permutation-supported candidate markers. Both models used identical training and test samples. Genomic-relationship matrices were calculated using training-group allele frequencies and standardized marker genotypes; training-monomorphic markers were excluded. The intercept and genetic and residual variance components were estimated by restricted maximum likelihood using training phenotypes. For the six size-adjusted traits, allometric regressions were refitted within each training fold and applied to the training and held-out fish. Prediction was summarized using pooled Pearson correlation, RMSE, Q^2^ relative to the training-fold mean baseline, and equal-group-weighted RMSE (Table S15b). Because candidate identification used the full discovery cohort, this marker-removal comparison was interpreted as exploratory.

Two previously prominent imputed signals, LG12:9370767:A:C and LG05:25769887:T:C, were examined retrospectively as diagnostic examples of discrepancies between original or imputed signals and recalled-genotype evidence. They were not predefined negative controls. Their diagnostic findings are reported in Section 3.3.

### 2.10. Candidate-Gene Annotation

Candidate windows extending 300 kb on either side of each marker were intersected with MAKER gene models [50]. The annotation contained 23,565 genes and 35,145 mRNA/protein models. Predicted proteins were searched against reviewed UniProtKB/Swiss-Prot with DIAMOND 2.2.7 [51,52]. Of 292 non-redundant genes in candidate windows, all had an extracted protein sequence, 114 had at least one Swiss-Prot hit, 105 had a passing high-confidence match, nine had low-confidence matches, and 178 had no reviewed-protein hit. Genes were prioritized by physical distance, alignment identity and coverage, annotation-edit distance, gene-model completeness, known developmental relevance, and consistency with local LD/conditional evidence. Proximity and homology were interpreted as prioritization evidence, not proof of causality (Table S11). Markers retained as family-supported candidates met the primary GWAS suggestive threshold of 1/M and a within-group permutation Benjamini–Hochberg-adjusted *p* value < 0.05 across the 18 selected tests. These numerical criteria defined association support. Functional gene prioritization was qualitative, considering proximity, protein homology, gene-model completeness, and relevant biological evidence, without assigning numerical functional tiers.

Public bighead-carp RNA-seq expression matrices were used to examine the expression of nominated candidate models. The dataset comprised 27 libraries from four BioProjects (PRJNA1253371, PRJNA721309, PRJNA1303128, and PRJNA453809), covering muscle, liver, intestine, hypothalamus–pituitary mixed tissue, and gill. Candidate annotations were matched to expression records using exact MAKER gene-model identifiers. Sample-level transcripts per million (TPM) were retained, and descriptive summaries were calculated separately for each BioProject, tissue, and source-study group. Multiple models sharing the same homology-based gene symbol were retained separately. Expression was interpreted as contextual evidence for candidate prioritization (Table S11).

### 2.11. Software and Reproducibility

Core analyses used bcftools and tabix [44,53], including the dosage, fill-tags, and setGT plugins; Beagle 5.5 [46,47]; PLINK 2.0 [48,49] (29 January 2025 build); GEMMA 0.98.5 [31]; R [54] with lme4 [40], Matrix, boot, ggplot2 [55], and patchwork; MAKER [50]; and DIAMOND 2.2.7 [51]. Random seeds and mask manifests were retained. The analysis archive contains per-step summaries, logs, truth manifests, candidate audits, figure-generation scripts, and the machine-readable supplementary tables required to reconstruct the reported counts. The GBLUP prediction analysis used Python 3.9.21 with NumPy 2.0.2 and SciPy 1.13.1.

## 3. Results

### 3.1. Phenotype Curation and Family Structure

The final phenotype set included 569 fish. Eleven of more than 4500 potential trait measurements were masked after multivariate consistency checks, preserving each fish for traits unaffected by a flagged value. BW remained right-skewed after raw-scale inspection, supporting logarithmic transformation, while allometric residuals separated local morphology from overall size (Figure S4; Table S1). The principal sample, marker, and validation checkpoints are summarized in Table 1.

**Figure 1:**
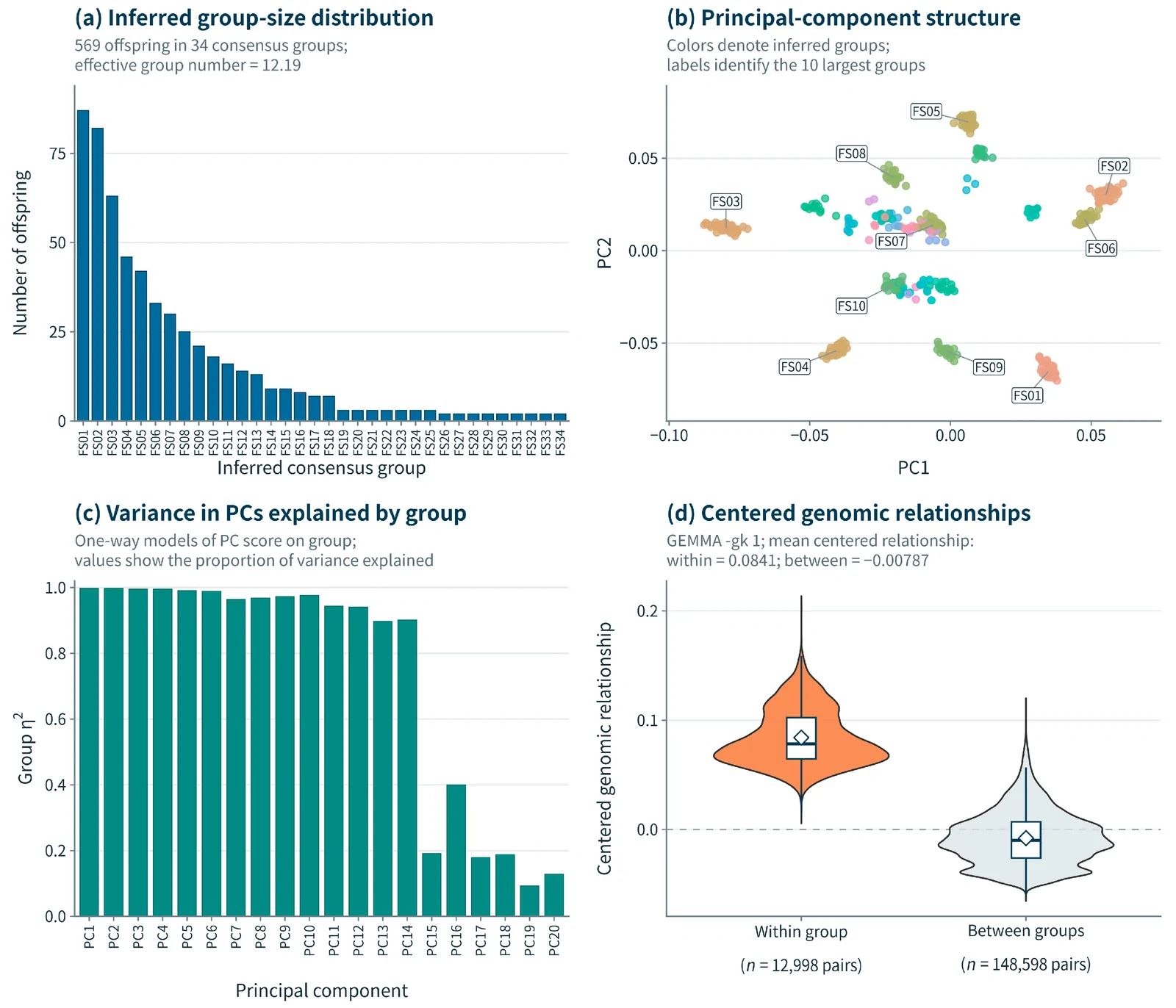
Inferred group structure and genomic relationships in the 569-offspring analysis population. (**a**) Numbers of offspring in the 34 inferred consensus full-sib groups. (**b**) PC1–PC2 scores colored by consensus group; labels identify the ten largest groups. (**c**) Proportion of variance in PC1–PC20 explained by group membership (η^2^). (**d**) Distributions of off-diagonal entries from the centered genomic-relationship matrix calculated using GEMMA -gk 1 for within-group (*n* = 12,998 pairs) and between-group (*n* = 148,598 pairs) comparisons. Diamonds denote means; boxes show medians and interquartile ranges. Matrix entries are sample-relative genomic relationships and are not pedigree-based kinship coefficients.

The 569 fish were distributed among 34 inferred consensus full-sib groups (Figure 1a,b; Table S2). Group sizes were highly unequal, ranging from two to eighty-seven offspring, with an effective group number of 12.19. Genetic structure was correspondingly strong: group membership explained approximately 90–100% of the variance in PC1–PC14 (η^2^ = 0.998 for PC1 and 0.901 for PC14; Figure 1c). The centered genomic-relationship matrix had a mean diagonal of 0.2717 and mean off-diagonal entries of 0.0841 within groups and −0.00787 between groups (Figure 1d). The standardized diagnostic matrix had a mean diagonal of 1.0000 and corresponding means of 0.2868 and −0.0270. Both matrices showed greater genomic similarity within groups than between groups. These sample-relative summaries describe the cohort structure but do not independently establish pedigree relationship classes.

Complementary-panel reconstruction showed high agreement with the original grouping. The E02 model assigned 565 offspring to 31 groups, with an adjusted Rand index of 0.99887 relative to the original grouping among assigned offspring; four offspring lacked two inferred parental assignments. The RAD model assigned all 569 offspring to 30 groups, with an adjusted Rand index of 0.99720. The largest groups were retained, whereas discrepancies involved several small groups. Across the corresponding candidate sensitivity analyses, all 18 effect directions and the same 17 FDR-supported tests were retained. On the matched 565-offspring sample set, changing from the original grouping to the E02 grouping altered the family fixed-effect coefficients by at most 5.22% relative to the original coefficients. Under the RAD grouping, the maximum corresponding change was 21.34%; three tests improved from moderately robust to robust in the LOFO assessment, and no classification deteriorated (Table S12).

Pairwise relationship likelihoods provided additional support for the inferred sibships. Using the complementary observed-marker panel, full siblings were the highest-supported relationship with a log10-likelihood-ratio margin ≥ 0.5 over the best alternative for 11,892 of 12,998 within-group pairs (91.49%) under E02 and 12,526 pairs (96.37%) under RAD. The corresponding proportions among between-group pairs were 1.08% and 2.29%. These pairwise classifications support the overall sibship structure, while cross-group support and changes involving small groups indicate residual assignment uncertainty.

Candidate-effect estimates were stable when size and inferred group effects were estimated jointly in one-stage models. All 18 trait–marker combinations retained their effect direction. The maximum absolute relative coefficient change was 4.20% across all combinations and 1.91% among the 17 originally permutation-supported combinations. The same 17 combinations passed BH-adjusted parametric *p* < 0.05. The LG01:24185345 association with logHW_adjBW remained nonsignificant (*p* = 0.165). These comparisons used identical observed-genotype samples and assess sensitivity of the selected candidate effects to the phenotype-model specification.

### 3.2. Family Contributions to Phenotypic Variation

Family random effects were significant after FDR correction for logBW (ICC = 0.207), logBL (ICC = 0.189), and logHW adjusted for logBW (ICC = 0.100). The remaining adjusted morphology traits had small, estimated ICCs (0–0.023) and did not pass FDR < 0.05. Thus, overall size and relative head width showed the clearest family-level component, whereas most other allometric traits varied predominantly within families. Full variance-component estimates and bootstrap results are provided in Table 2.

### 3.3. BAM Recall and Imputation Validation

Recall across all 24 linkage groups reproduced every target coordinate and reference allele. The median per-sample mean depth across all targets was 4.74 (IQR: 4.02–5.85; range: 2.94–18.66), and the median per-site mean depth was 4.66 (IQR: 3.96–5.97; range: 0–39.78). Among the 35,450 sites without a passing genotype, 35,398 contained only reference or missing raw calls and lacked FORMAT/GQ. Only 27 sites had no positive recorded depth. Across 77,760 downsampled reference-cell evaluations, the original DP ≥ 2/GQ ≥ 10/allele-balance ≥ 0.20 filters retained 72.35% of evaluations, with 97.86% concordance among retained calls. For reference heterozygotes, retained-call concordance increased from 79.94% at DP = 2 to 87.67% at DP = 3 and 94.96% at DP = 4–5. The DP ≥ 4/GQ ≥ 20 and DP ≥ 6/GQ ≥ 30 filters increased overall concordance to 99.71% and 99.95%, respectively, while reducing retention to 41.67% and 20.12% (Table S4). After cell-level DP/GQ/allele-balance masking, 32,724, 32,417, and 30,095 SNPs passed MAF ≥ 0.05 at call-rate thresholds of 0.50, 0.60, and 0.70. All three Beagle scenarios imputed every masked validation cell.

The three call-rate thresholds produced similar common-mask imputation performance. Overall concordance was 94.13%, 94.17%, and 94.11% for CALL50, CALL60, and CALL70, respectively. CALL60 had marginally higher overall, non-reference, and heterozygote concordance, whereas CALL50 had slightly higher balanced accuracy (Table 3). CALL60 was retained as a practical compromise between marker retention and imputation performance, retaining 32,417 SNPs compared with 32,724 for CALL50 and 30,095 for CALL70. These small differences do not establish a substantial accuracy advantage for CALL60.

Genotype- and MAF-stratified comparisons showed that no threshold performed best across all classes (Table S5b,c; Figure S2). For CALL60, concordance was 87.42%, 93.11%, 96.14%, and 95.95% in the reference-depth strata DP 4–5, 6–9, 10–19, and ≥20, respectively. Corresponding heterozygote concordance was 87.42%, 88.66%, 90.35%, and 90.97%. Because the DP 4–5 stratum contained only heterozygous reference genotypes, overall depth-stratified differences also reflected genotype composition. Results stratified jointly by depth and genotype are provided in Table S5e.

Post hoc examination of two previously prominent imputed signals identified discrepancies with recalled-genotype evidence. LG12:9370767:A:C was all-reference after targeted BAM recall (AC = 0) and showed strong base-quality bias (BQBZ = −17.2); it was excluded from further candidate analysis. For LG05:25769887:T:C, the association in the source-observed subset was weaker (likelihood-ratio *p* = 0.0172), and targeted masking recovered 70.7% of genotypes overall and 58.3% of heterozygotes. This locus was therefore not retained as a primary candidate (Figure S6).

### 3.4. Primary GWAS and Candidate Validation

**Figure 2:**
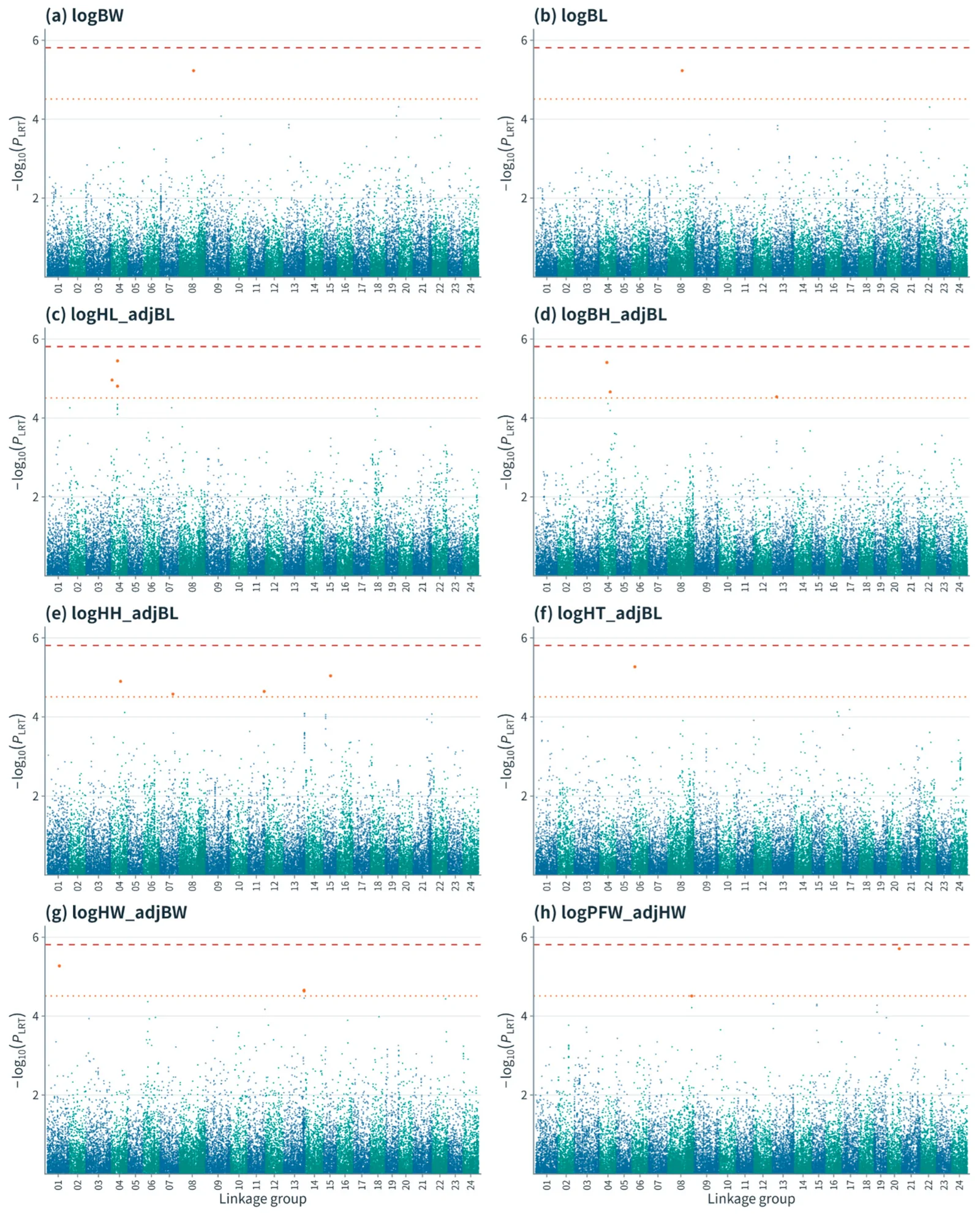
Manhattan plots for the primary PC0 GWAS using recalled genotype inputs before Beagle imputation. Missing marker genotypes were replaced by SNP-specific means in GEMMA, and the global centered genomic-relationship matrix was estimated from the LD-pruned Beagle-imputed dataset. (**a**) logBW, (**b**) logBL, (**c**) logHL_adjBL, (**d**) logBH_adjBL, (**e**) logHH_adjBL, (**f**) logHT_adjBL, (**g**) logHW_adjBW, and (**h**) logPFW_adjHW. Alternating colors distinguish the 24 linkage groups. Red dashed lines show trait-specific Bonferroni thresholds (0.05/M), orange dotted lines show suggestive thresholds (1/M), and orange points exceed the suggestive threshold. No association in these primary PC0 scans met the trait-specific Bonferroni threshold.

The eight primary PC0 scans using recalled genotype inputs tested 32,295–32,417 SNPs per trait, with 565–569 individuals contributing available phenotypes. Eighteen trait–marker combinations met the trait-specific suggestive threshold of approximately *p*_LRT_ < 3.1 × 10^−5^. No association met the trait-specific Bonferroni threshold of approximately 1.55 × 10^−6^. Genomic inflation factors ranged from 1.006 to 1.187 (Figure 2; Figure S1; Table S6b,c). Separately, the comparative Beagle-imputed PC0/PC3/PC5 grid comprised 24 analyses, each testing 32,290–32,304 SNPs (Table S6a).

LOCO analyses retained the same tested-marker sets as the primary scans. All 18 original candidate associations retained their effect directions, with a maximum absolute relative-coefficient change of 2.07%; 17 retained suggestive support. For logPFW_adjHW, LG08:47756139:G:A shifted from *p*_LRT_ = 3.091 × 10^−5^ to 3.492 × 10^−5^ despite an essentially unchanged effect estimate. Genome-wide LOCO scans identified twenty-four suggestive associations, including seventeen original candidates and seven additional associations. None met the trait-specific Bonferroni threshold. Genomic inflation factors ranged from 1.080 to 1.194 (Table S13).

In the recalled-input PC sensitivity analyses, genomic inflation factors ranged from 0.997 to 1.440 under PC3 and from 0.968 to 1.183 under PC5. PC adjustment reduced inflation for logPFW_adjHW but did not uniformly improve calibration. All 18 original candidates retained their effect directions, with maximum absolute relative coefficient changes of 15.21% under PC3 and 11.92% under PC5. Sixteen associations retained suggestive support under PC3, 14 under PC5, and 14 across all three specifications. Both logPFW_adjHW associations lost suggestive support under PC3 and PC5, whereas the linked LG13 logHW_adjBW associations lost support only under PC5. Three original candidates met the trait-specific Bonferroni threshold under each alternative model (Table S14).

**Figure 3:**
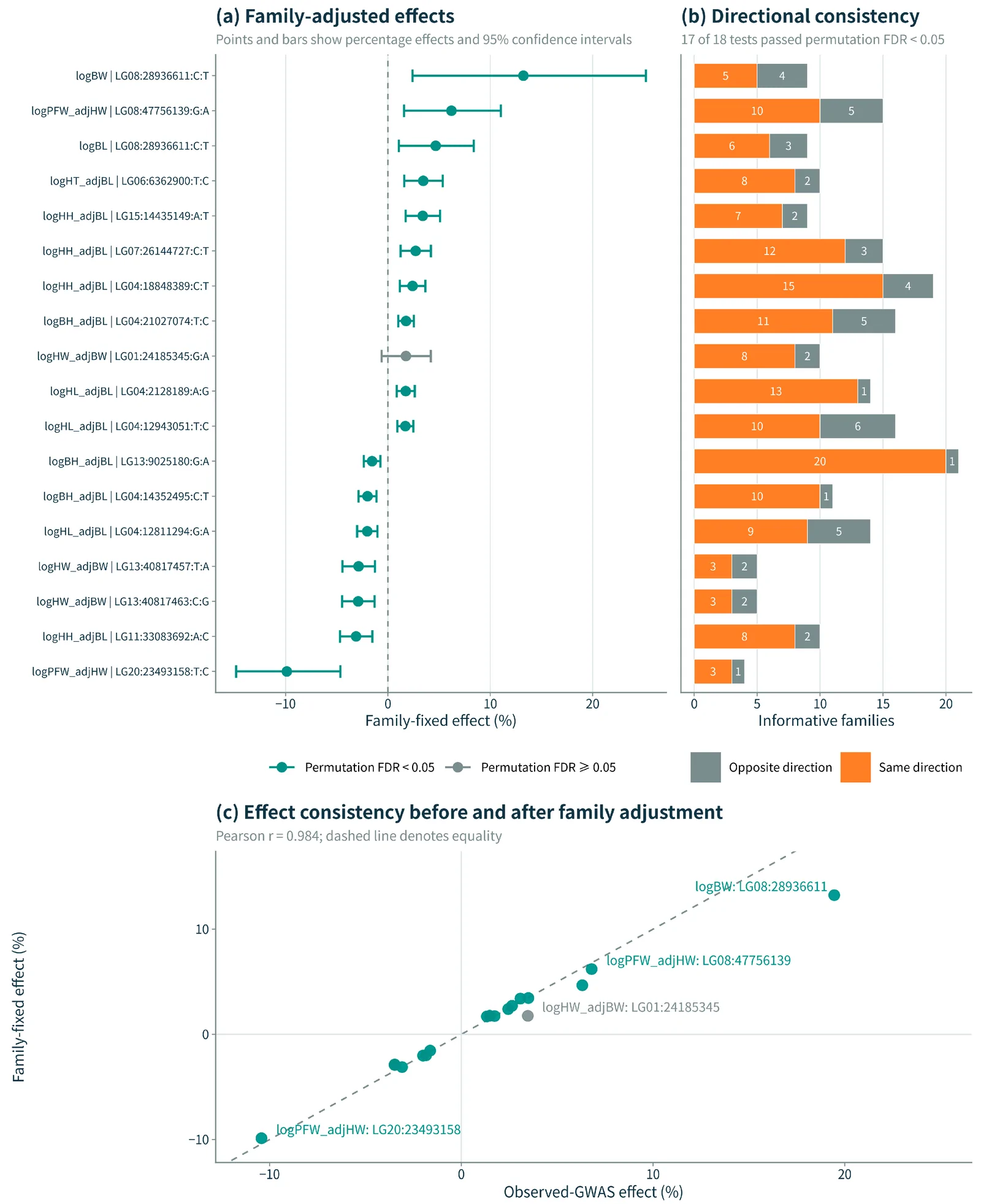
Family-aware validation of 18 suggestive trait–marker tests. (**a**) Family-fixed allelic effects, expressed as percentages, with 95% confidence intervals; color denotes whether the within-family permutation test passed FDR < 0.05. (**b**) Numbers of informative families with effects in the same or opposite direction as the overall association. (**c**) Agreement between observed-GWAS and family-fixed percentage effects; the dashed line denotes equality (Pearson *r* = 0.984). Seventeen tests had BH-adjusted permutation *p* values < 0.05 across the 18 selected candidates; this adjustment did not account for selection from the genome-wide scan. Family-fixed effects, group-specific effects, and permutation results were calculated from actual observed candidate genotypes and available phenotypes. The GWAS effects compared in panel (**c**) were obtained from the primary GEMMA scans using recalled genotype inputs with SNP-mean replacement of missing calls.

Eighteen trait–marker combinations selected from the primary GWAS underwent candidate-level assessment using observed genotypes. Seventeen had BH-adjusted *p* values < 0.05 across these 18 tests after 10,000 within-group permutations (Figure 3; Table S8). This adjustment was restricted to the selected candidates and did not account for their selection from the preceding genome-wide scan. LG01:24185345:G:A for logHW_adjBW was not supported by the permutation test (*p* = 0.115) and was excluded from the retained candidate set. The 17 supported combinations represented 16 markers. LG13:40817457:T:A and LG13:40817463:C:G were in complete dosage LD (*r*^2^ = 1) and were treated as one linked candidate region. LG08:28936611:C:T was associated with both logBW and logBL. The candidate associations and their technical and within-group support are summarized in Table 4.

Additional group-level analyses distinguished directional consistency from statistical support (Table 5; Table S8). Exact sign tests remained significant after BH adjustment for logHL_adjBL at LG04:2128189:A:G and logBH_adjBL at LG13:9025180:G:A, with adjusted *p* values of 0.0165 and 3.78 × 10^−4^, respectively. No heterogeneity test remained significant after adjustment. In 100 complementary half-group splits, 13 of the 18 tests retained matching effect directions in every split, although simultaneous nominal support in both halves varied among candidates. For LG08:28936611:C:T, directions matched in 88/100 splits for logBW and 89/100 for logBL, but neither test achieved two-sided *p* < 0.05 in both halves of any split. For LG20:23493158:T:C, directions matched in 56/88 evaluable splits; the remaining 12 splits were not estimable in both halves.

The six LG04 candidates spanned 18.90 Mb. Across all 15 marker pairs, observed-dosage r^2^ ranged from 0.000009 to 0.315 and absolute D′ from 0.009 to 0.659. Among 139 fish from 23 inferred groups with observed genotypes at all six markers, statistical phasing identified 25 haplotype configurations. Four had frequencies ≥5% and occurred in 16, three, seven, and five groups, respectively. Group-adjusted omnibus haplotype tests yielded nominal *p* values of 0.266 for logHL_adjBL, 0.0176 for logBH_adjBL, and 0.0181 for logHH_adjBL. In six-marker conditional models using 138–139 complete-case fish, individual-marker *p* values ranged from 0.071 to 0.814. These findings did not support a single strongly linked block spanning the six markers, but did not establish independent effects at each candidate position (Table S10).

Leave-one-family-out analysis classified ten trait–marker combinations as robust, seven as moderately robust, and the LG20:23493158:T:C association with logPFW_adjHW as family-sensitive (Table S9). The LG20 marker segregated within only four families and is therefore retained as exploratory despite a significant within-family permutation test. Observed call rates for most retained candidates were 0.70–0.94; LG04:14352495:C:T had a call rate of 0.687 (Figure S3; Table S7). Family-specific effect patterns across all 34 families are shown in Figure S5. Incremental PVE after accounting for inferred consensus group ranged from 1.09% to 4.98% across the 17 permutation-supported trait–marker tests. The LG08:28936611:C:T marker explained an additional 1.09% and 1.23% of phenotypic variance for logBW and logBL, respectively. The largest estimate was 4.98% for LG04:21027074:T:C and logBH_adjBL (Table S15a).

In leave-one-consensus-group-out prediction, correlations for the complete-panel GBLUP ranged from −0.0105 to 0.2715 across the eight traits. Removing the trait-specific supported candidate markers produced similar prediction performance for most traits, with absolute correlation differences below 0.001 for seven traits. The largest difference occurred for logBH_adjBL, for which the complete-panel model achieved a correlation of 0.1020 compared with 0.0690 after candidate removal. Corresponding RMSE values were 0.037072 and 0.037249, representing a relative reduction of 0.47%. Thus, the marker-removal comparison showed limited incremental predictive contribution from the specified candidate markers under this evaluation design (Table S15b).

### 3.5. Functional Prioritization

**Figure 4:**
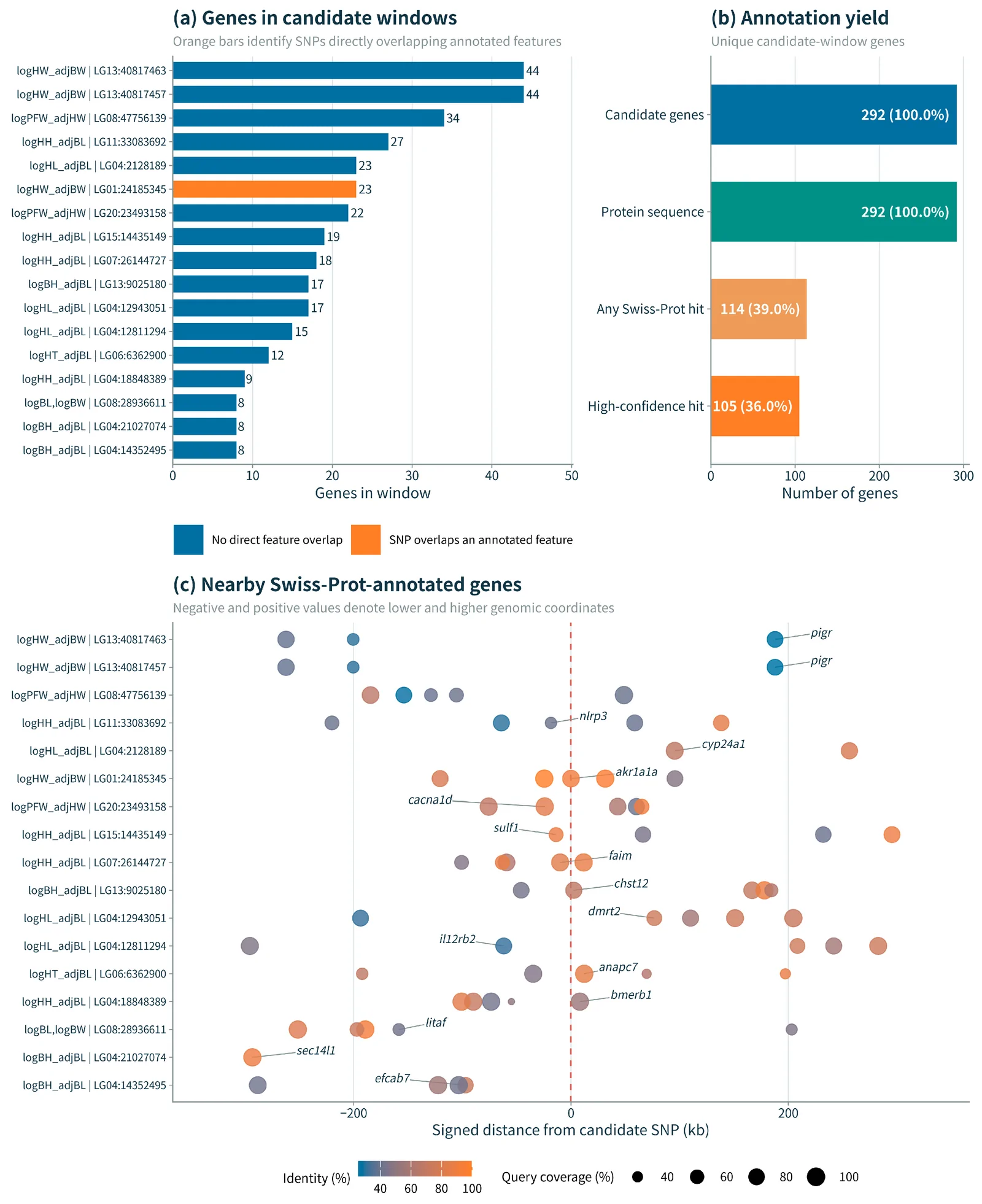
Candidate-window structural and functional annotation. (**a**) Numbers of MAKER genes within ±300 kb of each of the 17 unique candidate markers; orange identifies markers that directly overlap an annotated feature. (**b**) Annotation yield among 292 non-redundant candidate-window genes: protein sequence availability, any reviewed Swiss-Prot hit, and a passing high-confidence hit. (**c**) Positions of up to five nearby Swiss-Prot-annotated genes per candidate marker. Signed distance indicates lower or higher genomic coordinates; color represents amino-acid identity and point size represents query coverage. Labels identify the closest functionally annotated gene for each marker.

The annotation supported a compact set of biologically interpretable genes, but gene-model quality varied (Figure 4; Table S11). Prioritized candidates included putative *dmrt2* near LG04:12943051, *cyp24a1* near LG04:2128189, *bmerb1*/*gadd45a* near LG04:18848389, *efcab7* near LG04:14352495, *anapc7* near LG06:6362900, *faim* near LG07:26144727, *chst12*/*gna12* near LG13:9025180, and *sulf1* near LG15:14435149.

Additional candidate regions included the second LG04 head-length signal near *il12rb2* and *dmrt2*, the weakly annotated *sec14l1* region, the cross-trait LG08:28936611 signal, the LG08:47756139 region containing *ctsk*, the LG11 region containing *nlrp3*, and the collapsed LG13:40817457/40817463 signal near *pigr*/*cd300* genes. The LG20 locus was annotated near a fragmented putative *cacna1d* model. Its association remained exploratory because it was family-sensitive and showed limited within-group segregation. These gene assignments are functional priorities based on proximity and homology rather than causal identifications.

All 25 nominated MAKER models, representing 20 homology-based gene symbols, were matched to the public expression matrices (Table S11). The *dmrt2*-annotated model was detected in both muscle groups, with mean TPM values of 6.76 and 4.49 in the high- and low-growth groups, respectively. The *cyp24a1*-annotated model was detected in liver across all three source-study groups, with mean TPM values of 489.08, 1972.01, and 1955.04. The *sulf1*- and *chst12*-annotated models were detected in hypothalamus-pituitary and gill samples. Expression was also detected for both *efcab7*-annotated models and both fragmented *cacna1d*-annotated models. Sample-level TPM values and the nine source-specific group summaries are provided in Table S11.

## 4. Discussion

### 4.1. Genotype Reconstruction as an Evidential Layer

Genotype reliability in low-depth GBS depends on both allele representation and the handling of uncertain calls. RAD/GBS allele dropout can introduce nonrandom missingness and genotyping error [21,22,23], while imputation draws information from haplotypes and related individuals [27,56,57]. The downsampling benchmark showed that low-depth heterozygotes remained particularly vulnerable to homozygous miscalls. At DP = 2, an allele-balance filter has limited discriminatory power when only one allele is sampled. Stronger DP/GQ thresholds improved concordance but substantially reduced retention, illustrating the trade-off between genotype reliability and available information.

Common-mask validation supported CALL60 as a practical compromise rather than demonstrating a substantial accuracy advantage over the other panels. Its aggregate performance nevertheless could not determine the reliability of every focal association. The LG12 audit identified a signal unsupported by reference-consistent reads, whereas the LG05 audit identified a polymorphic marker whose association was not sustained convincingly by observed-genotype evidence. These contrasting outcomes show why candidate evaluation benefits from combining matrix-level recovery assessment with locus-specific read evidence and association tests based on actual observed genotypes.

### 4.2. Family Structure and Within-Cohort Candidate Evaluation

The factorial mating design created groups with shared parental backgrounds. Because broodstock genotypes and individual identifiers were unavailable, the inferred groups could not be matched to verified crosses or used to resolve all half-sib connections. Within-group tests and group-omission analyses therefore assess internal support and sensitivity within this cohort; holding out one group does not provide replication across independent parental backgrounds.

The close correspondence between principal components and inferred groups indicates that relatedness dominated the cohort structure. A genomic-relationship matrix models polygenic covariance, but markers from the tested linkage group may also absorb local association effects through proximal contamination [32,33,34,35]. LOCO analyses showed limited changes in candidate coefficients, although one borderline association lost suggestive support and genomic inflation was not reduced. Within-group permutation addresses a different question by testing genotype–phenotype correspondence after removing group means [36]. The LG01 association retained suggestive support under LOCO but failed within-group permutation, illustrating why stability across covariance specifications alone is insufficient.

PC sensitivity similarly distinguished stability of effect direction from stability of statistical support. PC adjustment weakened the logPFW_adjHW associations and reduced support for the linked LG13 logHW_adjBW markers under PC5. This attenuation could reflect reduced confounding or a change in the genetic contrast being estimated; the present analyses do not distinguish these explanations. Associations sensitive to model specification therefore require particular attention in subsequent replication.

The distribution of segregating genotypes among groups also affects the breadth of support. Many offspring from one cross can estimate a within-cross contrast precisely without demonstrating transferability to other parental backgrounds. The LG20 association illustrates this limitation because only four groups were informative. Future mapping populations would benefit from additional independent parental backgrounds and explicit reporting of informative-group sizes, genotype distributions, and shared-parent relationships. 

### 4.3. Allometry, Developmental Modules, and Candidate-Gene Interpretation

The prominence of adjusted head and body dimensions highlights the distinction between size and shape. Log-scale allometric adjustment shifts the phenotype toward morphological variation conditional on the selected size covariate [37,38]. Associations with these residual traits may therefore concern developmental timing, skeletal patterning, extracellular-matrix organization, or tissue-specific growth rather than general growth alone. The LG04 associations involved several morphological traits and did not form a single strongly linked block spanning all six markers. However, the six-marker conditional analysis did not resolve independent marker effects, leaving the number and identity of underlying causal variants uncertain.

Fish developmental studies provide specific biological hypotheses for several candidate regions. In zebrafish, *dmrt2a*/*terra* contributes to bilateral synchronization of the segmentation clock [58], while *sulf1* modulates BMP and FGF signaling during somite and horizontal myoseptum development [59]. Zebrafish *cyp24a1* responds to vitamin D through a feedback pathway involved in calcium homeostasis [60], and *chst12a* is expressed in developing head structures and pharyngeal cartilage [61]. These findings support investigating the nearby bighead carp homologous models, but do not establish that they mediate the associated morphological variation. The focal markers lie 76,674 bp from the *dmrt2* model, 13,687 bp from *sulf1*, 95,470 bp from *cyp24a1*, and 2585 bp from *chst12* (Table S11). Physical proximity and developmental relevance therefore provide complementary grounds for prioritization, rather than evidence of a causal marker–gene relationship.

Previous fish-mapping studies provide a useful comparison for these hypotheses. QTL mapping of growth traits in bighead carp nominated genes associated with cell proliferation, including *sash1* [9], while a head-size GWAS identified regions containing proliferation- and bone-development-related genes, including *prdm1b* and *bmp8a* [8]. In common carp, combined GWAS and QTL mapping implicated IGF signaling and genes including *igf1*, *igf3*, *notch2*, and *sfrp2* in head-size variation [12]. The present candidates suggest additional hypotheses involving extracellular-matrix modification, developmental patterning, and mineral regulation. These comparisons concern biological processes; they do not establish replication of the same genes or homologous QTL, which would require orthology and coordinate harmonization across studies.

Public expression data provide biological context for candidate prioritization. The *dmrt2*-annotated model was detected in muscle, *cyp24a1* in liver, and *sulf1* and *chst12* in hypothalamus–pituitary and gill samples. Published bighead-carp transcriptomes from skeletal tissues and the palatal organ provide additional resources for investigating these candidates [14,62]. Expression should nevertheless be linked to the exact annotated model and does not establish a marker–gene mechanism. This distinction is particularly important for the fragmented *efcab7*- and *cacna1d*-annotated models and the linked LG13 region near *pigr*/*cd300* genes. These regions remain locus-level candidates pending improved gene models, regional fine mapping, and functional investigation.

### 4.4. From Association Evidence to Breeding Decisions

The conditional PVE estimates and group-held-out prediction results provide complementary measures of breeding relevance. Incremental PVE of 1.09%–4.98% describes the improvement in phenotypic fit associated with individual candidate markers in the discovery cohort. The prediction analysis evaluates whether retaining those markers improves prediction for fish from held-out consensus groups. Although the complete-panel model performed better for logBH_adjBL, its relative RMSE reduction was only 0.47%, and differences were very small for most other traits. These findings support a cautious assessment of the candidates’ incremental predictive value in the current genomic panel.

Genomic prediction provides a broader strategy for evaluating marker information for polygenic production traits [4,5,6,17,63]. The present candidates offer priorities for replication, targeted genotyping, and functional investigation. Their use in breeding programs will require confirmation of effect direction, linkage phase, and predictive contribution across additional genetic backgrounds, year classes, and production environments [15,16,64,65,66]. The current study therefore contributes a set of genomic hypotheses and quantitative evidence that can guide further work toward genetic improvement of growth and body conformation in bighead carp.

### 4.5. Limitations and a Validation Agenda

Several limitations constrain inference. Uncertainty in small, inferred groups and unequal group sizes limit the breadth of evidence for some candidates. Sex was not determined, so potential sex-associated variation in growth and morphology could not be evaluated. Group-level phenotypic differences may also include maternal effects and other shared non-genetic influences and should not be interpreted as purely genetic. Hard-genotype imputation does not propagate posterior uncertainty into downstream analyses [27,56,57], and the physical-distance approximation used for phasing limits interpretation of recombination. Reduced-representation sequencing may incompletely tag causal variants, while fragmented gene models constrain effector-gene assignment. Independent population replication and functional testing remain necessary to establish transferability and biological mechanisms.

A staged validation program follows directly from these constraints. The next statistical step is targeted, high-depth genotyping of the focal regions in more independent families and year classes, coupled to prospective tests of effect direction and heterogeneity. A linkage map and denser regional sequencing would then separate correlated markers and refine credible intervals. RNA sequencing or spatial expression assays in relevant skeletal, cartilage, muscle, and growth tissues could nominate effector genes, followed by allele-specific expression or functional perturbation where feasible. Such a sequence of replication, fine mapping, molecular prioritization, and functional testing is more likely to establish causality than adding further post hoc annotations to the same association panel [64,65,66].

## 5. Conclusions

This study identified suggestive associations for growth and allometrically adjusted body-shape traits in bighead carp. Within-group permutation supported 17 of 18 selected trait–marker associations, involving 16 unique markers, while annotation and public expression data provided biological context for candidate prioritization. Group-held-out prediction indicated limited incremental contribution from the specified candidate markers for most traits. These findings provide focused targets for independent replication, functional investigation, and evaluation across breeding populations and generations, supporting further research toward genetic improvement and new-variety development in bighead carp.

## Figures and Tables

**Table 1:** Overview of samples, variants, and validation checkpoints.

Stage	Samples	Sites/Cells	Key Result
Initial candidate VCF	590	89,807 SNPs	24 LGs plus 34 non-LG records
Reference-consistent LG set	590	89,684 SNPs	6955 REF/ALT swaps corrected; 89 non-simple sites excluded
Family-aware phenotype set	569	34 families	Trait *n* = 565–569 after 11 cell-level masks
BAM recall after cell masks	569	89,684 SNP targets	53,935 polymorphic; overall call rate 0.490
MAF05 + CALL60 candidates	569	32,417 SNPs	Selected by common-mask validation
Common validation mask	569	274,566 cells	Truth: DP ≥ 4, GQ ≥ 30; 5% stratified mask
Final imputed matrix	569	32,304 SNPs	0 missing; MAF ≥ 0.05
LD-pruned structure set	569	9086 SNPs	PCA20 and global genomic kinship

**Table 2:** Bootstrap family-variance components for transformed phenotypes.

Phenotype	*n*	ICC	95% CI	Bootstrap *p*	FDR	FDR < 0.05
logBW	569	0.2066	0.0792–0.3288	0.00050	0.00133	Yes
logBL	567	0.1895	0.0750–0.3023	0.00050	0.00133	Yes
logHL_adjBL	565	0.0193	0–0.0645	0.04698	0.07516	No
logBH_adjBL	566	0.0061	0–0.0365	0.23488	0.26844	No
logHH_adjBL	565	<0.0001	~0	0.62319	0.62319	No
logHT_adjBL	565	0.0233	0–0.0739	0.02899	0.05797	No
logHW_adjBW	568	0.1001	0.0223–0.1860	0.00050	0.00133	Yes
logPFW_adjHW	567	0.0130	0–0.0494	0.06247	0.08329	No

**Table 3:** Common-mask imputation performance across pre-imputation site-call-rate thresholds.

Set	SNPs	Truth Cells	Concord.	Non-Ref Concord.	Het Concord.	Balanced Acc.	Dosage *r*^2^	Kappa
CALL50	32,724	274,566	0.9413	0.9022	0.8898	0.9538	0.8575	0.8969
CALL60	32,417	274,566	0.9417	0.9026	0.8904	0.9537	0.8588	0.8976
CALL70	30,095	274,566	0.9411	0.9015	0.8893	0.9529	0.8572	0.8964

**Table 4:** Suggestive associations with within-group support and nearby annotated genes.

**A** **. Association Results**						
**Marker**	**Trait**	**Call Rate**	**MAF**	**p_LRT_**	**Perm.p**	**Candidate Gene(s)**
LG04:12943051:T:C	logHL_adjBL	0.789	0.300	3.56 × 10^−6^	1.00 × 10^−4^	*dmrt2*
LG04:14352495:C:T	logBH_adjBL	0.687	0.174	3.91 × 10^−6^	1.00 × 10^−4^	*efcab7*
LG15:14435149:A:T	logHH_adjBL	0.863	0.056	9.11 × 10^−6^	1.00 × 10^−4^	*sulf1*
LG04:21027074:T:C	logBH_adjBL	0.705	0.166	2.18 × 10^−5^	1.00 × 10^−4^	*sec14l1*
LG04:2128189:A:G	logHL_adjBL	0.835	0.123	1.09 × 10^−5^	2.00 × 10^−4^	*cyp24a1*
LG04:12811294:G:A	logHL_adjBL	0.786	0.083	1.56 × 10^−5^	2.00 × 10^−4^	*il12rb2; dmrt2*
LG13:40817463:C:G	logHW_adjBW	0.856	0.071	2.20 × 10^−5^	2.00 × 10^−4^	*pigr/cd300 cluster*
LG11:33083692:A:C	logHH_adjBL	0.772	0.069	2.26 × 10^−5^	2.00 × 10^−4^	*nlrp3/rnh1*
LG13:9025180:G:A	logBH_adjBL	0.787	0.144	2.91 × 10^−5^	3.00 × 10^−4^	*chst12; gna12*
LG13:40817457:T:A	logHW_adjBW	0.854	0.071	2.31 × 10^−5^	4.00 × 10^−4^	*pigr/cd300 cluster*
LG04:18848389:C:T	logHH_adjBL	0.873	0.131	1.26 × 10^−5^	7.00 × 10^−4^	*bmerb1; gadd45a*
LG07:26144727:C:T	logHH_adjBL	0.822	0.122	2.63 × 10^−5^	1.40 × 10^−3^	*faim*
LG06:6362900:T:C	logHT_adjBL	0.898	0.054	5.38 × 10^−6^	2.30 × 10^−3^	*anapc7*
LG20:23493158:T:C	logPFW_adjHW	0.944	0.051	1.96 × 10^−6^	2.80 × 10^−3^	*putative cacna1d*
LG08:28936611:C:T	logBL	0.826	0.069	5.89 × 10^−6^	8.50 × 10^−3^	*litaf*
LG08:28936611:C:T	logBW	0.826	0.069	5.90 × 10^−6^	1.17 × 10^−2^	*litaf*
LG08:47756139:G:A	logPFW_adjHW	0.770	0.312	3.09 × 10^−5^	9.10 × 10^−3^	*ctsk; asparaginase-like*
**B. Observed Genotype Quality and Pairwise Linkage Disequilibrium**						
**Marker**	**Genotype Counts 0/0; 0/1; 1/1**	**Median DP**	**Informative Groups**	**LD Partner**	**|D′|**	**Paired Fish**
LG04:12943051:T:C	220; 189; 40	8	16	LG04:12811294:G:A	0.659	356
LG04:14352495:C:T	261; 124; 6	5	11	LG04:12943051:T:C	0.283	320
LG15:14435149:A:T	441; 45; 5	5	9	—	—	—
LG04:21027074:T:C	291; 87; 23	10	16	LG04:18848389:C:T	0.532	353
LG04:2128189:A:G	360; 113; 2	5	14	LG04:12811294:G:A	0.272	373
LG04:12811294:G:A	0; 74; 373	4	14	LG04:12943051:T:C	0.659	356
LG13:40817463:C:G	418; 69; 0	6	5	LG13:40817457:T:A	1.000	486
LG11:33083692:A:C	378; 61; 0	6	10	—	—	—
LG13:9025180:G:A	319; 129; 0	4	21	LG13:40817457:T:A	0.126	381
LG13:40817457:T:A	417; 69; 0	6	5	LG13:40817463:C:G	1.000	486
LG04:18848389:C:T	369; 126; 2	6	19	LG04:21027074:T:C	0.532	353
LG07:26144727:C:T	354; 114; 0	5	15	—	—	—
LG06:6362900:T:C	457; 53; 1	6	10	—	—	—
LG20:23493158:T:C	482; 55; 0	6	4	—	—	—
LG08:28936611:C:T	407; 61; 2	5	9	LG08:47756139:G:A	0.020	370
LG08:47756139:G:A	170; 263; 5	12	15	LG08:28936611:C:T	0.020	370

*p*_LRT_ values are from the primary GEMMA PC0 scans using recalled genotype inputs with SNP-specific mean replacement of missing calls. Permutation *p* values were obtained from actual observed candidate genotypes and available phenotypes, with genotypes shuffled within inferred consensus groups. Call rate is the proportion of the 569 fish with an actual observed genotype. The two LG13 markers at 40,817,457 and 40,817,463 bp were treated as one linked candidate region. Genotype counts represent actual observed REF/REF (0/0), REF/ALT (0/1), and ALT/ALT (1/1) calls among the 569 fish. Median DP was calculated among these calls. Informative-group counts are trait specific and require available phenotypes and within-group variation in observed genotype dosage; the count for LG08:28936611:C:T is nine for both logBL and logBW. Absolute D′ was calculated for the named, physically nearest retained candidate on the same linkage group using Beagle-derived phases in fish with observed genotypes at both markers and matching observed and phased dosages. “Paired fish” gives the corresponding sample count. A dash indicates that no same-linkage-group partner was available. These pairwise estimates do not establish a chromosome-wide haplotype block.

**Table 5:** Group-level direction, heterogeneity, and internal half-group effect stability for the 18 candidate trait–marker tests.

Trait	Marker	Same: Opposite Groups	Heterogeneity F (df_1_, df_2_)	Heterogeneity *p*	Sign-Test *p* (BH-Adjusted *p*)	Direction Agreement	Both Halves *p* < 0.05
logPFW_adjHW	LG20:23493158:T:C	3:1	2.058 (3, 498)	0.1050	0.6250 (0.7500)	56/88	13/88
logHL_adjBL	LG04:12943051:T:C	10:6	1.442 (15, 396)	0.1245	0.4545 (0.6293)	100/100	64/100
logBH_adjBL	LG04:14352495:C:T	10:1	0.457 (10, 345)	0.9167	0.0117 (0.0703)	100/100	100/100
logHT_adjBL	LG06:6362900:T:C	8:2	0.959 (9, 465)	0.4737	0.1094 (0.2461)	100/100	30/100
logHL_adjBL	LG04:2128189:A:G	13:1	0.321 (13, 424)	0.9887	0.0018 (0.0165)	100/100	83/100
logHH_adjBL	LG04:18848389:C:T	15:4	1.212 (18, 440)	0.2467	0.0192 (0.0864)	100/100	78/100
logBH_adjBL	LG04:21027074:T:C	11:5	0.905 (15, 355)	0.5583	0.2101 (0.3782)	100/100	70/100
logBH_adjBL	LG13:9025180:G:A	20:1	0.517 (20, 391)	0.9594	2.10 × 10^−5^ (3.78 × 10^−4^)	100/100	85/100
logPFW_adjHW	LG08:47756139:G:A	10:5	1.288 (14, 387)	0.2117	0.3018 (0.4938)	79/100	1/100
logHW_adjBW	LG01:24185345:G:A	8:2	0.759 (9, 461)	0.6542	0.1094 (0.2461)	91/100	0/100
logBL	LG08:28936611:C:T	6:3	0.767 (8, 426)	0.6325	0.5078 (0.6529)	89/100	0/100
logBW	LG08:28936611:C:T	5:4	0.711 (8, 428)	0.6819	1.0000 (1.0000)	88/100	0/100
logHH_adjBL	LG15:14435149:A:T	7:2	0.389 (8, 444)	0.9265	0.1797 (0.3594)	100/100	60/100
logHL_adjBL	LG04:12811294:G:A	9:5	1.210 (13, 396)	0.2692	0.4240 (0.6293)	100/100	16/100
logHW_adjBW	LG13:40817463:C:G	3:2	0.867 (4, 448)	0.4835	1.0000 (1.0000)	100/100	56/100
logHH_adjBL	LG11:33083692:A:C	8:2	0.303 (9, 394)	0.9735	0.1094 (0.2461)	100/100	67/100
logHW_adjBW	LG13:40817457:T:A	3:2	0.863 (4, 447)	0.4862	1.0000 (1.0000)	100/100	56/100
logHH_adjBL	LG07:26144727:C:T	12:3	1.783 (14, 416)	0.0388	0.0352 (0.1266)	100/100	47/100

Direction agreement and simultaneous nominal support are reported as counts among evaluable splits. “Both halves *p* < 0.05” refers to two-sided coefficient tests in both halves. Heterogeneity *p* values are unadjusted; BH-adjusted sign-test *p* values are shown in parentheses.

## Data Availability

The raw sequencing data for this study have been deposited in the NCBI Sequence Read Archive database under BioProject accession number PRJNA1535785.

## Supplementary Materials

The following supporting information can be downloaded at: https://www.mdpi.com/article/10.3390/ani16203271/s1. The supplementary package comprises Figures S1–S6 and Tables S1–S15. Figure S1: Quantile–quantile plots for the primary recalled-input PC0 GWAS; Figure S2: Common-mask imputation validation; Figure S3: Candidate-marker technical-quality control; Figure S4: Transformed phenotype distributions; Figure S5: Group-specific candidate effects; Figure S6: LG05 imputation audit. Table S1: Phenotype curation and allometric transformation; Table S2: Consensus group assignments; Table S3: Reference-allele harmonization; Table S4: Recalled-marker retention, depth distributions, and downsampling assessment; Table S5: Common-mask imputation validation, including depth-stratified results; Table S6: Primary and comparative GWAS summaries; Table S7: Candidate-marker technical audits; Table S8: Group-adjusted effects, permutation tests, and group-level stability assessments; Table S9: Leave-one-group-out sensitivity; Table S10: Pairwise LD, haplotype, and conditional analyses; Table S11: Candidate annotation and public transcriptome expression; Table S12: Complementary-panel grouping and candidate sensitivity; Table S13: LOCO GWAS sensitivity; Table S14: Recalled-input PC covariate sensitivity; Table S15: Candidate incremental PVE and group-held-out genomic prediction.

## Abbreviations

The following abbreviations are used in this manuscript:

AC	Alternate-allele count
AD	Allelic depth
ADF	Allelic depth on the forward strand
ADR	Allelic depth on the reverse strand
AF	Alternate-allele frequency
ALT	Alternate allele
AN	Total number of called alleles
ARI	Adjusted Rand index
BAM	Binary Alignment/Map format
BH	Body height
BL	Body length
BQBZ	Mann–Whitney U z-score for base-quality bias between REF and ALT alleles
BWA-MEM2	Accelerated implementation of the Burrows–Wheeler Aligner MEM algorithm
BW	Body weight
CI	Confidence interval
DNA	Deoxyribonucleic acid
DP	Read depth
DS	Genotype dosage
EM	Expectation–maximization
FDR	False discovery rate
GBS	Genotyping-by-sequencing
GEMMA	Genome-wide Efficient Mixed-Model Association
GBLUP	Genomic best linear unbiased prediction
GP	Genotype posterior probability
GQ	Genotype quality
GRM	Genomic-relationship matrix
GT	Genotype
GWAS	Genome-wide association study
HH	Head height
HL	Head length
HT	Head thickness
HW	Head width
ICC	Intraclass correlation coefficient
IQR	Interquartile range
LD	Linkage disequilibrium
LG	Linkage group
LOCO	Leave-one-chromosome-out
LOFO	Leave-one-family-out
LRT	Likelihood-ratio test
MAE	Mean absolute error
MAD	Median absolute deviation
MAF	Minor-allele frequency
NCBI	National Center for Biotechnology Information
PC	Principal component
PCR	Polymerase chain reaction
PE	Paired-end
PFW	Palatal-organ weight
PVE	Phenotypic variance explained
Q–Q	Quantile–quantile
QC	Quality control
RAD	Restriction-site-associated DNA
REF	Reference allele
RMSE	Root-mean-square error
RNA	Ribonucleic acid
SNP	Single-nucleotide polymorphism
SP	Phred-scaled strand-bias p value
SRA	Sequence Read Archive
SSE	Residual sum of squares
SST	Total sum of squares
TPM	Transcripts per million
VCF	Variant Call Format
