# Supplementary material for: Family-Aware Genome-Wide Association Mapping of Growth and Body-Shape Traits in Bighead Carp (Hypophthalmichthys nobilis)
Source: Animals (Basel). 2026 Oct 17;16(20):3271. doi: 10.3390/ani16203271 (PMC13700713; doi:10.3390/ani16203271)
Supplement: Supplementary file 1 [file animals-16-03271-s001.zip › Supplementary Figures.pdf]

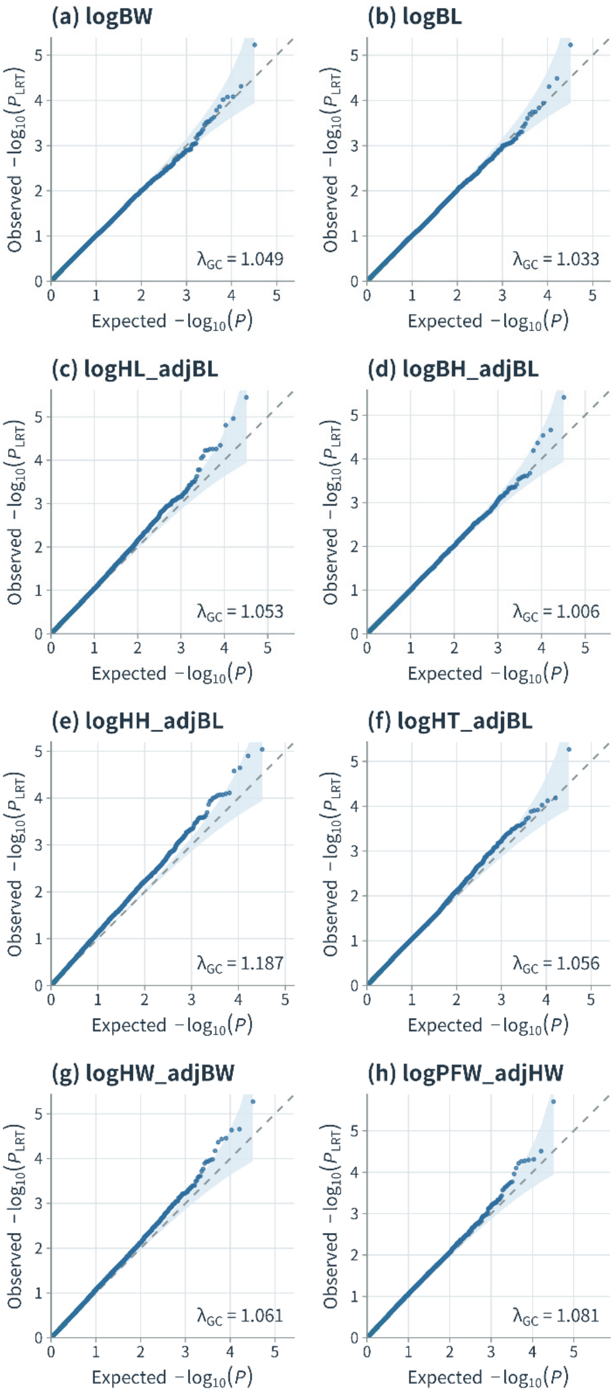

Figure S1. Quantile–quantile plots for the primary recalled-input PC0 GWAS. Panels (a–h) correspond to logBW, logBL, logHL_adjBL, logBH_adjBL, logHH_adjBL, logHT_adjBL, logHW_adjBW, and logPFW_adjHW. Points show observed versus expected $-\log_{10}(P_{LRT})$; dashed lines denote equality and shaded regions are 95% confidence envelopes. Each panel reports the genomic inflation factor (λ_{GC}).

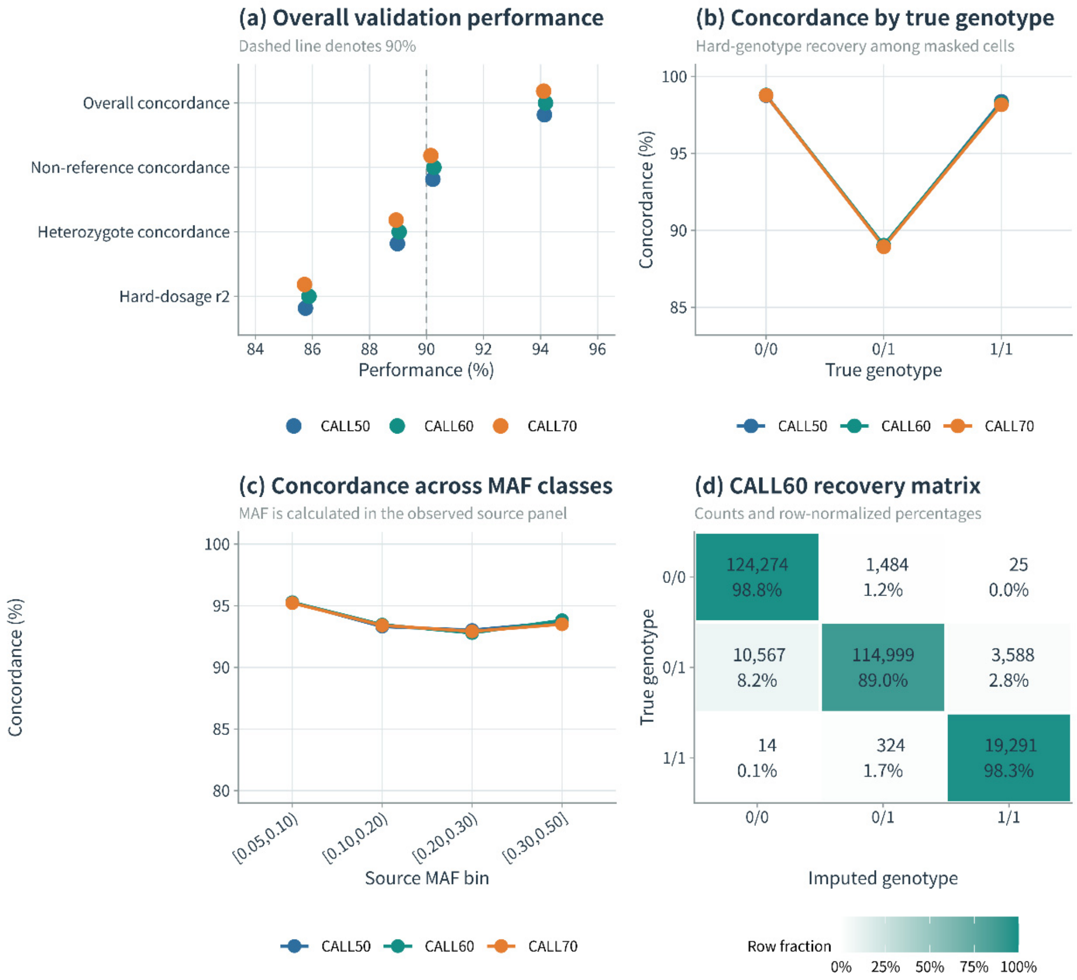

Figure S2. Common-mask imputation validation for CALL50, CALL60, and CALL70. (a) Overall concordance, non-reference concordance, heterozygote concordance, and hard-dosage r^2; the dashed line marks 90%. (b) Hard-genotype concordance by true genotype. (c) Concordance across source-panel MAF bins. (d) CALL60 recovery matrix showing counts and row-normalized percentages for 274,566 masked truth cells.

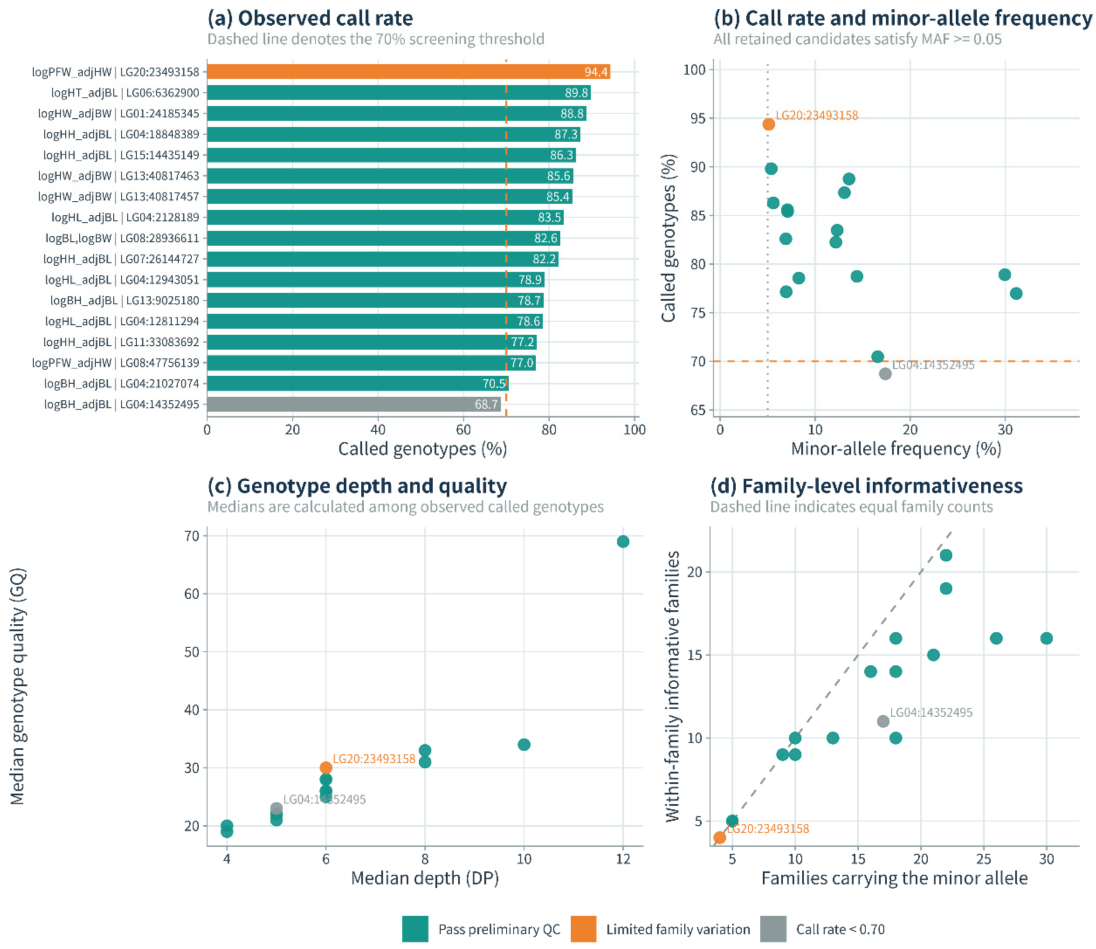

Figure S3. Technical quality control for 17 unique observed candidate markers. (a) Observed call rates with the 70% screening threshold. (b) Call rate versus MAF; all retained candidates have MAF ≥ 0.05. (c) Median genotype depth and genotype quality among observed calls. (d) Families carrying the minor allele versus families informative for within-family analysis. Colors distinguish preliminary pass, limited family variation, and call rate < 0.70.

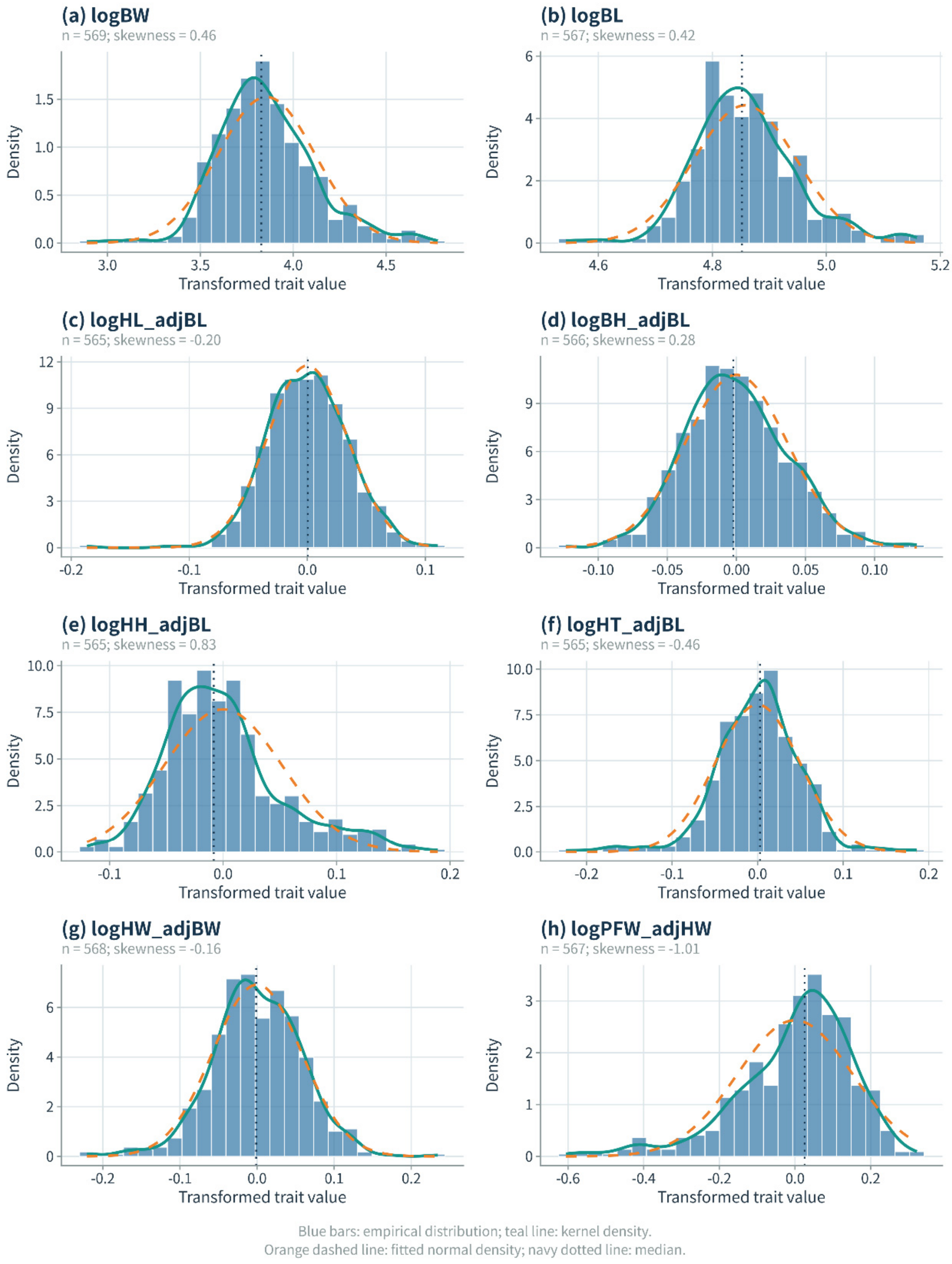

Figure S4. Distributions of the eight transformed GWAS phenotypes. Panels show (a) logBW, (b) logBL, (c) logHL_adjBL, (d) logBH_adjBL, (e) logHH_adjBL, (f) logHT_adjBL, (g) logHW_adjBW, and (h) logPFW_adjHW. Blue bars show empirical distributions, teal curves show kernel densities, orange dashed curves show fitted normal densities, and navy dotted lines show medians. Analysed sample size and skewness are reported in each panel.

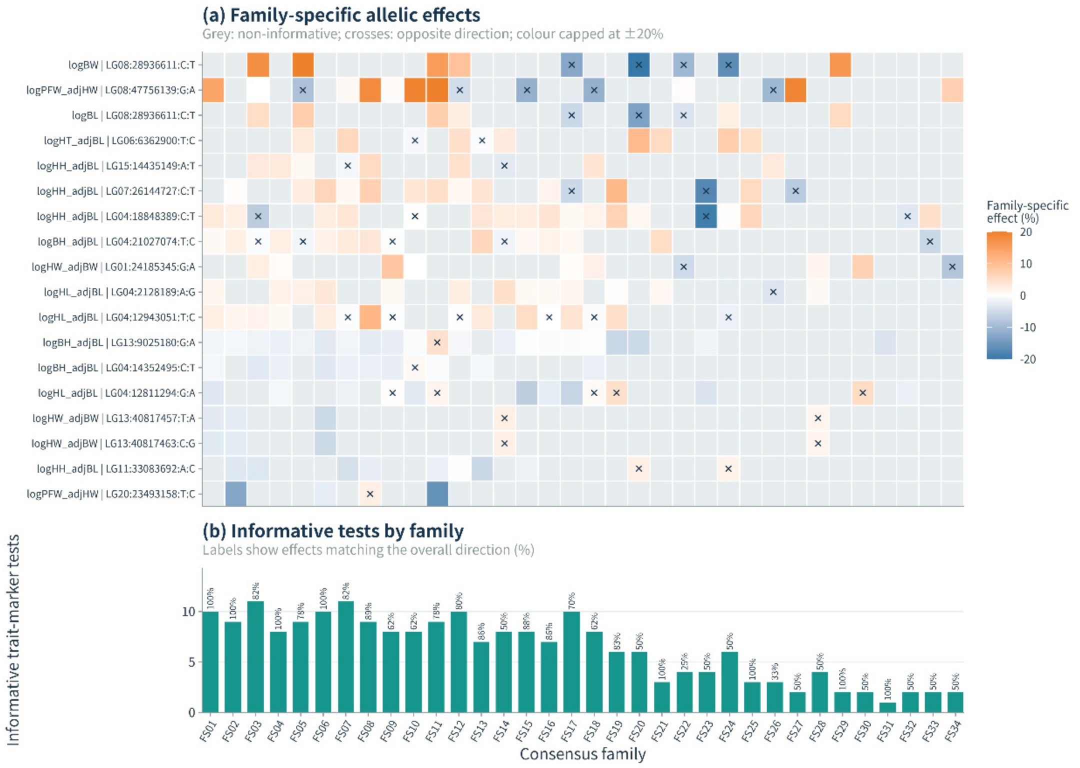

Figure S5. Family-specific allelic effects for the 18 candidate trait–marker tests. (a) Heat map of percentage effects across 34 inferred consensus families. Grey cells are non-informative, crosses mark effects opposite to the overall association, and the colour scale is capped at ±20%. (b) Number of informative tests per family; labels give the percentage of informative effects matching the overall direction.

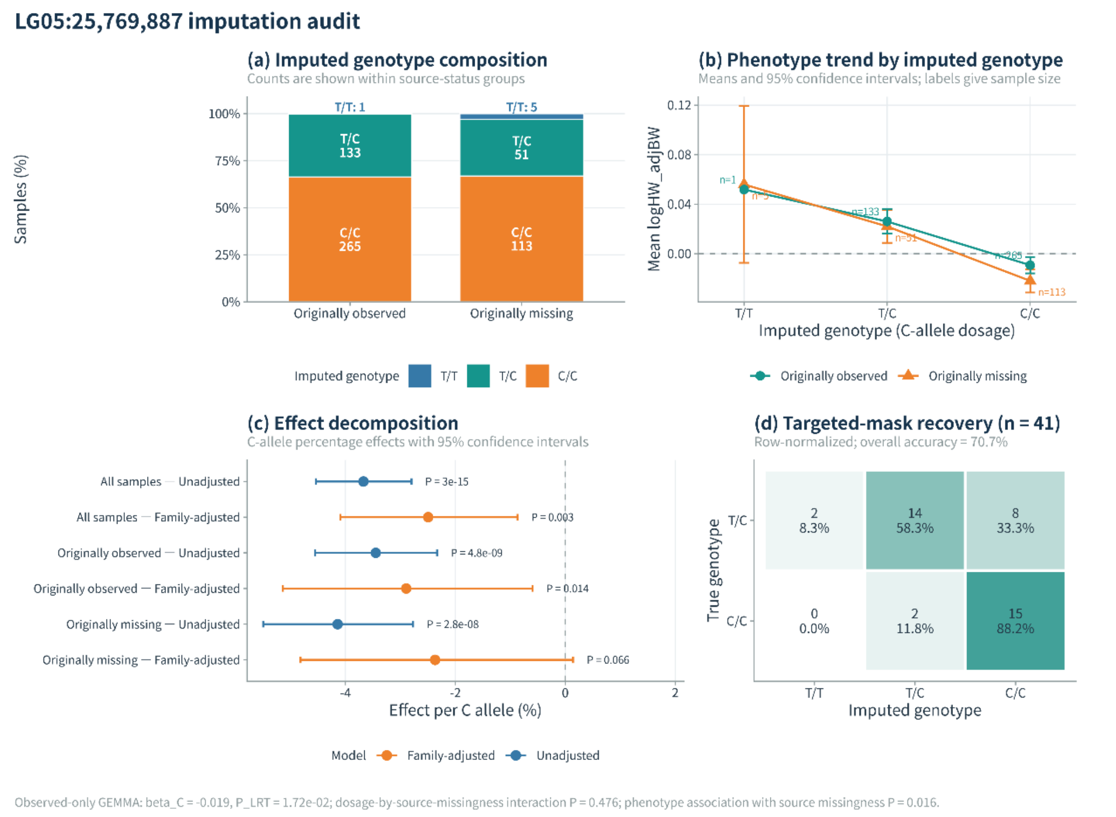

Figure S6. Targeted audit of the imputation-dependent LG05:25,769,887 signal for logHW_adjBW. (a) Imputed-genotype composition among samples with originally observed or missing source genotypes. (b) Phenotype means and 95% confidence intervals by imputed genotype and source-missingness group. (c) Unadjusted and family-adjusted C-allele effects for all samples and source-observed/source-missing subsets. (d) Row-normalized recovery matrix for 41 targeted masked cells (overall accuracy, 70.7%). The observed-only GEMMA association was weaker ($\beta_C = -0.01898$; $P_{LRT} = 0.0172$), the dosage-by-missingness interaction was not significant ($P = 0.476$), and source missingness was associated with the phenotype ($P = 0.016$).
